# A Natural Alkaloid, 6-Hydroxymethyldihydronitidine, Suppresses Tumor Progression by Co-Regulating Apoptosis, Ferroptosis, and FAK Pathways

**DOI:** 10.3390/biom15060814

**Published:** 2025-06-04

**Authors:** Haojing Jiang, Jiantong Hou, Jianliang Wang, Jing Xu, Yuanqiang Guo

**Affiliations:** State Key Laboratory of Medicinal Chemical Biology, College of Pharmacy, Nankai University, Tianjin 300350, China; 2120231659@mail.nankai.edu.cn (H.J.); 2120211292@mail.nankai.edu.cn (J.H.); 2120221569@mail.nankai.edu.cn (J.W.)

**Keywords:** alkaloid, anti-tumor, apoptosis, ferroptosis, FAK signaling pathway

## Abstract

Cancer treatment remains a formidable challenge globally. Natural products, particularly natural alkaloids, have emerged as significant resources for the development of novel anti-tumor drugs due to their structural diversity and unique biological activities. Our team previously isolated an alkaloid, 6-hydroxymethyldihydrochelerythrine (6-HMDN), from *Zanthoxylum ailanthoides*. Subsequent in vitro and in vivo activity screenings, utilizing cell-based assays and a zebrafish xenograft model, revealed that 6-HMDN significantly inhibited the proliferation of HepG2 and MCF7 cells and effectively suppressed HepG2 cell migration. Mechanistic studies indicated that 6-HMDN induced tumor cell apoptosis by modulating the Bcl-2/Bax protein balance and activating the caspase cascade. Furthermore, 6-HMDN augmented intracellular reactive oxygen species (ROS) levels, thereby promoting ferroptosis, as evidenced by lipid ROS accumulation and glutathione (GSH) depletion. Additionally, 6-HMDN attenuated focal adhesion kinase (FAK) phosphorylation, leading to the inhibition of tumor cell migration. In vivo experiments further substantiated the capacity of 6-HMDN to effectively suppress tumor proliferation and metastasis. These findings demonstrate that 6-HMDN exhibits potent anti-tumor activity, exerting its effects through multiple mechanisms involving the regulation of apoptosis, ferroptosis, and the FAK signaling pathway. Therefore, 6-HMDN may be considered a promising candidate for anti-tumor drug development.

## 1. Introduction

Cancer constitutes a leading global health crisis [1], imposing substantial economic and psychological burdens on patients and their families. Modern medicine has achieved notable advancements in cancer treatment, encompassing surgery, radiotherapy, targeted therapies, and immunotherapy [2]. Traditional chemotherapeutic agents, although effective in eliminating tumor cells, are often associated with severe adverse effects, including bone marrow suppression, nausea, vomiting, etc., significantly diminishing patients’ quality of life. Moreover, drug resistance in tumor cells significantly hinders successful treatment outcomes [3]. Consequently, the development of novel chemotherapeutic drugs characterized by enhanced efficacy, reduced toxicity, and the ability to circumvent drug resistance is of paramount importance.

Tumor progression involves hallmark processes such as uncontrolled proliferation and metastasis [4]. In recent years, apoptosis (programmed cell death) and ferroptosis (iron-dependent lipid peroxidation-driven cell death) have become key targets for tumor therapy. The abnormal activation of anti-apoptotic proteins disrupts normal cell apoptosis, leading to the transformation into immortal cancer cells [5]. Therefore, inducing cell apoptosis has become a core strategy in cancer treatment. With the development of various chemotherapy drugs, drug-resistant tumors are increasingly appearing, and ferroptosis provides a unique mechanism to overcome treatment resistance [6]. Ferroptosis selectively eliminates cells with high lipid peroxidation sensitivity, bypassing the pathways involved in traditional drug resistance. The dual targeting of these mechanisms, such as inducing cell apoptosis while disrupting antioxidant defense, has the potential to develop more effective multimodal cancer therapies.

Natural products have long served as an invaluable source of innovative pharmaceuticals, particularly in the realm of anti-cancer drug discovery. Their unique chemical structures, diverse biological activities, and often lower toxicity have garnered considerable attention from researchers [4]. Alkaloids, a large class of nitrogen-containing organic compounds derived primarily from plants and microorganisms, have demonstrated significant pharmacological potential [7,8]. In cancer therapy research, various alkaloid compounds exert anti-tumor effects through diverse mechanisms, including the induction of apoptosis [9] and ferroptosis [10,11], modulation of cellular redox status, and cell cycle interference. For instance, vincristine, an alkaloid isolated from *Catharanthus roseus*, is a classic chemotherapeutic agent that induces apoptosis by inhibiting microtubule polymerization [12]. Berberine, another alkaloid, has been shown to induce ferroptosis and exhibits broad-spectrum anti-tumor activity [10]. The application of alkaloids in cancer treatment has sparked our strong interest in natural alkaloids. Our research group has previously isolated and characterized 6-hydroxymethyldihydronitidine (6-HMDN), a benzophenanthridine alkaloid, from *Zanthoxylum ailanthoides*, a traditional medicinal plant. Existing studies have demonstrated that the medicinal plant *Z. ailanthoides* possesses a wide range of biological activities, including antioxidant [11], anti-HIV [13], and anti-inflammatory [14] properties. These medicinal functions are closely associated with its chemical constituents, which have been reported to include aromatic glucosides, coumarins, sterols, sesquiterpenes, alkaloids, and lignans [13,15,16]. Among these constituents, alkaloids are regarded as the primary functional components. However, research on the anti-tumor effects of the alkaloid 6-HMDN has not yet been reported.

Given the unmet need for multi-targeted cancer therapies, this study investigated the anti-tumor potential and mechanisms of 6-HMDN, leveraging many reported alkaloids’ known roles in inducing apoptosis, ferroptosis, and regulating oxidative stress. Using in vitro cell-based assays and in vivo zebrafish experiments, we assessed 6-HMDN’s effects on tumor cell proliferation and migration. We hypothesized that 6-HMDN exerts anti-tumor activity via multiple key pathways, including modulation of apoptosis-related proteins, reactive oxygen species (ROS) levels, ferroptosis markers, and the phosphorylation status of focal adhesion kinase (FAK). This work establishes a foundation for developing 6-HMDN as a novel, effective, and low-toxicity anti-cancer agent.

## 2. Materials and Methods

### 2.1. Materials and Cell Culture

The human hepatocellular carcinoma cell line HepG2, the human breast cancer cell line MCF7, and the human embryonic kidney 293T cell line HEK-293T were obtained from the Shanghai Institute of Life Sciences, Chinese Academy of Sciences (Shanghai, China). Cells were maintained in Dulbecco’s Modified Eagle Medium (DMEM), supplemented with 10% fetal bovine serum (FBS) and 1% penicillin–streptomycin, and cultured in a humidified incubator at 37 °C with 5% CO_2_. Detailed information regarding materials is provided in the Supplementary Materials.

### 2.2. Extraction and Purification of 6-HMDN

The plant *Z. ailanthoides* was purchased from the Anguo Traditional Chinese Medicine Market in Hebei Province in June 2023 and stored properly after the identification. The extraction, separation, and purification of 6-HMDN mainly followed the experimental methods of the research group in the early stage [17]. The dried bark of the plant (20.0 kg) was refluxed three times with 95% methanol to obtain a residue, which was then refluxed under reduced pressure to obtain a crude extract (2.2 kg). Afterwards, 2.2 L of distilled water was added to dissolve the crude extract. The pH of the solution was adjusted to a range of 1.5–1.8 by adding hydrochloric acid. Then, ethyl acetate was added multiple times for extraction according to the volume ratio of the organic layer to the aqueous layer being 1:1. After the extraction, the pH of the aqueous layer was adjusted to a range of 9.5–10.0 with ammonia water. The extraction procedure was repeated. The organic phases were combined and concentrated, yielding 322.0 g of the alkaloid extract. The extract was transferred to a silica gel column (200–300 mesh) and eluted with a gradient of petroleum ether–acetone in different volume ratios (ranging from 100:0 to 100:31). Meanwhile, the elution process was monitored by thin-layer chromatography (TLC), resulting in seven fractions (Fr.1 to Fr.7). Subsequently, Fr.3 (10.5 g) was separated by medium-pressure liquid chromatography (MPLC) using an aqueous solution containing 82% methanol as the eluent, and one fraction (F3-1) was obtained. Finally, F3-1 was purified by preparative high-performance liquid chromatography (preparative HPLC, YMC-pack ODS-AM column, 20 mm × 250 mm) with an aqueous solution containing 78% methanol as the eluent, yielding a pale yellow powder (16.3 mg), which was identified as 6-HMDN. The extraction yield was calculated to be 0.0000815%.

### 2.3. MTT Assay

The MTT assay was employed to assess the cytotoxic effects of 6-HMDN on HepG2, MCF7, and HEK-293T cell lines. Cells were seeded in 96-well plates and incubated at 37 °C in a 5% CO_2_ atmosphere for 24 h. Subsequently, the cells were treated with varying concentrations of 6-HMDN and incubated for a further 48 h. Following incubation, 20 μL of MTT solution (5 mg/mL) was added to each well, and the cells were incubated for an additional 4 h under the same conditions. The culture medium was then removed, and 150 μL of DMSO was added to each well to dissolve the formazan crystals. Absorbance was measured at 492 nm using a microplate reader. Each experiment was performed in triplicate. The inhibition rate was calculated using the following formula:

Inhibition rate (%) = (OD_control_ − OD_experiment_)/OD_control_ × 100%

### 2.4. Zebrafish Husbandry

Adult AB strain wild-type zebrafish were procured from Feixi Biotechnology Co., Ltd. (Shanghai, China). Zebrafish were maintained in a recirculating aquaculture system equipped with aeration and ultraviolet sterilization. The water temperature was consistently maintained at 27.5 ± 1.0 °C. Zebrafish were fed twice daily and maintained under a 14 h light/10 h dark cycle.

### 2.5. In Vivo Anti-Tumor Assay Using a Zebrafish Xenograft Model

The zebrafish experimental protocol was approved by the Animal Ethics Committee of Nankai University. All zebrafish-related studies were conducted in accordance with the ARRIVE guidelines, and the zebrafish used in the study were not gender differentiated. For embryo collection, male and female zebrafish (2:1 ratio) were placed at opposite ends of a breeding tank and kept in an incubator at 28.5 °C overnight. The following day, after 30 min of light exposure, the partition was removed to allow for mating. Embryos were collected 30 min post-mating and cultured in sterile Holt buffer. At 2 days post-fertilization (dpf), chorions were removed using trypsin. At 3 dpf, larvae were anesthetized, and 5 nL of CM-DiI stained tumor cell suspension (1 × 10^7^ cells/mL) was injected into the yolk sac to establish the tumor xenograft model [18]. Six hours post-injection, zebrafish were treated with different concentrations of 6-HMDN (0.05, 0.1, and 0.2 μM) for 48 h. Afterwards, the 0.02% solution of tricaine was used for general anesthesia of zebrafish larvae. Tumor cell proliferation and metastasis were monitored using a laser confocal microscope (Leica, TCS SP8, Wetzlar, Germany) and quantified using ImageJ (1.51K; National Institute of Health, Bethesda, MD, USA, 2017).

### 2.6. Apoptosis Assay

Flow cytometry was utilized to detect apoptosis induced by 6-HMDN [19]. HepG2 cells in the exponential growth phase were seeded at a density of 1 × 10^5^ cells per well in 12-well plates. After 24 h, the cells were treated with varying concentrations of 6-HMDN (5, 10, and 20 μM). Following 48 h of treatment, cells from both the experimental and control groups were harvested, washed with PBS to remove impurities and media components, and resuspended in binding buffer. Annexin V-FITC and PI-staining solutions were added, gently mixed, and incubated for 15 min at room temperature in the dark. Subsequently, binding buffer was added to dilute the cell suspension. The analysis was performed using a BD flow cytometer (BD, Franklin Lakes, NJ, USA), and the apoptotic rate was determined by analyzing cell populations with different fluorescence intensities using FlowJo (10.0.7; FlowJo LLC, Ashland, OR, USA, 2014).

### 2.7. Measurement of ROS Production

Flow cytometry was employed to measure ROS production following 6-HMDN treatment [20]. Cells in the exponential growth phase were seeded in 12-well plates at a density of 1 × 10^5^ cells per well and incubated at 37 °C for 24 h. After 48 h of 6-HMDN treatment, the cells were collected and washed with PBS. The cells were then stained with the DCFH-DA probe, a cell-permeable fluorescent dye used to detect reactive oxygen species (ROS). Non-fluorescent DCFH-DA diffuses into cells, where it is hydrolyzed to DCFH and oxidized by ROS to form fluorescent DCF. The stained cells were washed and resuspended in serum-free DMEM. ROS generation was detected by flow cytometry and analyzed using FlowJo software.

### 2.8. Measurement of Glutathione Content

Intracellular glutathione (GSH) levels were quantified using a Micro Reduced GSH Detection Kit (Akkbine, Wuhan, China). HepG2 cells in the exponential growth phase were seeded in six-well plates at a density of 5 × 10^5^ cells per well and cultured for 24 h in a CO_2_ incubator. The cells were then treated with different concentrations (5, 10, and 20 μM) of 6-HMDN and erastin (30 μM) for 24 h. Following treatment, the cells were collected, resuspended in the kit-provided extraction buffer, and disrupted by sonication to extract protein samples. The total protein concentration was determined using the bicinchoninic acid (BCA) assay, a colorimetric technique for quantifying protein concentrations based on their interaction with copper ions in an alkaline environment. The GSH content was measured according to the manufacturer’s instructions, and the data were normalized to protein concentration.

### 2.9. Measurement of Lipid ROS Levels

Flow cytometry was used to quantify lipid ROS levels in HepG2 cells [21]. HepG2 cells were seeded in 12-well plates at a density of 1 × 10^5^ cells/mL (1 mL per well) and incubated for 24 h. After treatment with varying concentrations of 6-HMDN (5, 10, and 20 μM) for 24 h, the cells were collected and incubated with a diluted working solution of the BODIPY^581/591^ probe (GlpBio, Shanghai, China) in the dark for 15 min. The cells were then centrifuged, washed, resuspended in serum-free DMEM, filtered, and aliquoted into flow cytometry tubes for immediate analysis using a flow cytometer. Data were processed using FlowJo software.

### 2.10. Wound-Scratch Assay

The wound-healing assay was performed to evaluate the effect of 6-HMDN on tumor cell migration [22]. HepG2 cells were seeded in 6-well plates at a density of 5 × 10^5^ cells per well and incubated for 24 h until reaching confluence. A linear scratch wound was created in the cell monolayer using a sterile pipette tip, and the cells were treated with different concentrations of 6-HMDN (2.5, 5, and 10 μM). Cell migration was monitored, and images were captured at 0 and 48 h post-treatment using a microscope. Images were analyzed using ImageJ software to quantify the cell migration rate.

### 2.11. Western Blotting Analysis

Western blotting was conducted to assess changes in the expression levels of proteins associated with relevant signaling pathways. The cell culture and drug treatment procedures were as described above. Following 48 h of incubation, the cells were harvested and the total cellular proteins were extracted using RIPA lysis buffer. Protein concentration was determined using a BCA Protein Concentration Assay Kit (Beyotime, Shanghai, China) and strictly according to the manufacturer’s instructions. Extracted proteins were separated by 12% SDS-PAGE, transferred to polyvinylidene fluoride (PVDF) membranes, blocked, and incubated with primary and secondary antibodies. Protein bands were visualized using enhanced chemiluminescence (Beyotime, Shanghai, China) and detected using an imager. The optical density was quantified using ImageJ software, and data were normalized to loading controls.

### 2.12. Statistical Analysis

All experimental data were analyzed using GraphPad Prism 6.0 software (GraphPad Software, Inc., San Diego, CA, USA). Statistical comparisons were performed using one-way analysis of variance (ANOVA). A *p*-value less than 0.05 (*p* < 0.05) was considered statistically significant.

## 3. Results

### 3.1. Identification of 6-HMDN

**Figure 1:**
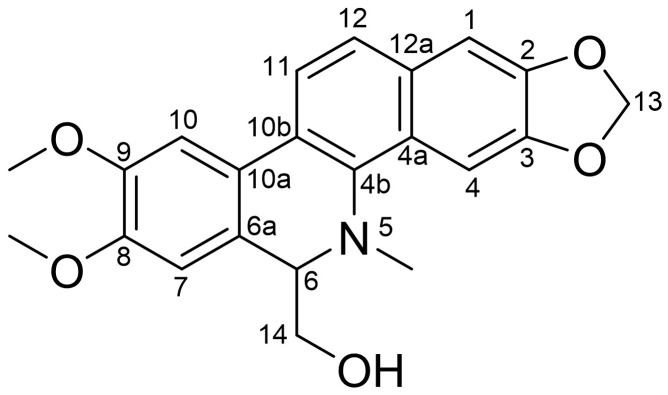
Chemical structure of 6-HMDN.

The purified compound was identified via nuclear magnetic resonance (NMR). By comprehensively analyzing its ^13^C NMR (CDCl_3_, 100 MHz) and ^1^H NMR data (CDCl_3_, 400 MHz) in Table 1, it was preliminarily inferred to be an alkaloid. After comparison with the reported data [23], it was conclusively identified as 6-HMDN. The chemical structure of 6-HMDN is shown in Figure 1.

### 3.2. 6-HMDN Inhibited Tumor Cell Proliferation In Vitro

To evaluate the cytotoxic potential of 6-HMDN, we assessed its effects on the following two human cancer cell lines: HepG2 (hepatocellular carcinoma) and MCF7 (breast cancer). 6-HMDN exhibited a more pronounced inhibitory effect on HepG2 cells. The half-maximal inhibitory concentration (IC_50_) of 6-HMDN against HepG2 cells was 8.15 ± 0.49 μM, while for MCF7 cells, it was 22.32 ± 2.21 μM. For comparison, the IC_50_ values of the positive control drug etoposide for HepG2 and MCF7 cells were 1.46 ± 0.06 μM and 17.10 ± 1.10 μM, respectively. These results indicate a differential sensitivity of the two cell lines to both 6-HMDN and etoposide. Notably, 6-HMDN demonstrated comparatively stronger cytotoxicity against HepG2 cells than MCF7 cells. Afterwards, we used the same method to determine the cytotoxicity of 6-HMDN in HEK-293T cells. The results show that the IC_50_ of 6-HMDN in HEK-293T cells was 46.78 ± 0.64 μM. After calculation, the selectivity indexes (SI) of 6-HMDN in HepG2 cells and MCF7 cells were 5.74 and 2.10, respectively, calculated as the ratio of IC_50_ (non-malignant tumor cells) to IC_50_ (tumor cells), which proved that 6-HMDN may specifically kill cancer cells [24].

### 3.3. 6-HMDN Inhibited Tumor Proliferation and Metastasis In Vivo

In vivo tumor progression is a complex process influenced by numerous factors [25]. We first conducted a zebrafish toxicity test. The zebrafish embryos in the experimental group developed normally, and there was no significant difference in the survival rate compared with that of the control group (Figure S1). This indicated that 6-HMDN had good biocompatibility and no toxic or side effects.

**Figure 2:**
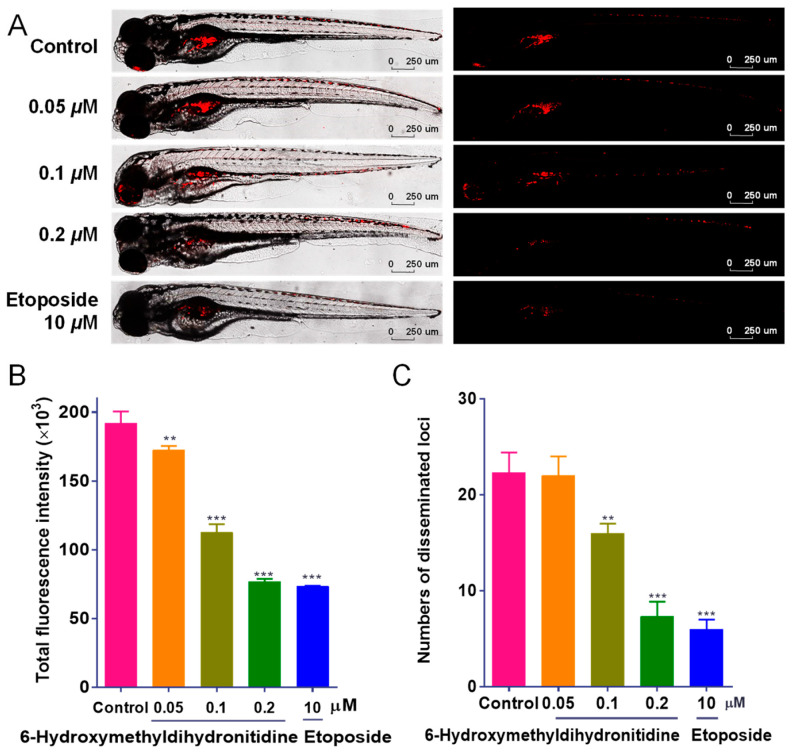
6-HMDN inhibited the proliferation and migration of HepG2 cells in the zebrafish xenograft model: (**A**) representative laser confocal microscopy images showing red fluorescence intensity and distribution in zebrafish; (**B**) quantification of HepG2 cell proliferation using ImageJ software; (**C**) quantification of HepG2 cell migration using ImageJ software. Data are presented as the mean ± SD. ** *p* < 0.01 and *** *p* < 0.001 compared with the control group, n = 3.

To further validate the anti-tumor efficacy of 6-HMDN in vivo, we utilized a zebrafish xenograft model. The model employs CM-DiI, a highly lipophilic fluorescent dye, which stably integrates into the cell membrane. Once incorporated, CM-DiI emits intense red fluorescence, allowing for long-term, non-invasive tracking of cells without significant cytotoxicity or interference with cell behavior. Its fluorescence persists through cell divisions, ensuring consistent visualization of cell lineages and migration patterns. Characterized by transparent embryos, it enables the real-time visualization of tumor dynamics through fluorescence labeling and recapitulates key aspects of the human tumor microenvironment, rendering it particularly useful for investigating tumor metastasis mechanisms. As depicted in Figure 2A, zebrafish in the control group exhibited intense red fluorescence signals and numerous metastatic foci, confirming the invasive nature of HepG2 cells. The clinical chemotherapeutic agent etoposide (10 μM) was included as a positive control, providing a benchmark for the experimental system’s reliability. Following 48 h of treatment with 6-HMDN, both the tumor fluorescence intensity and the number of metastatic foci were reduced in a dose-dependent manner, indicating that 6-HMDN effectively suppressed tumor proliferation and metastasis in vivo.

### 3.4. 6-HMDN Induced Apoptosis in HepG2 Cells

**Figure 3:**
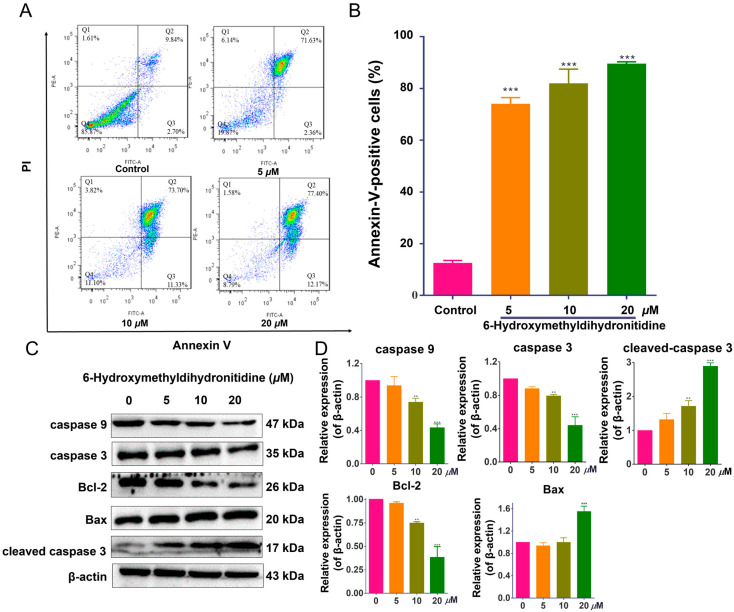
6-HMDN induced apoptosis in HepG2 cells and regulated the Bax/Bcl-2 signaling pathway and apoptosis-related proteins. HepG2 cells were treated with 6-HMDN at concentrations of 5, 10, and 20 μM for 48 h: (**A**) cells were stained with Annexin V-FITC and PI, and apoptotic cells were detected by flow cytometry; (**B**) histogram depicting the proportion of apoptotic HepG2 cells following 48 h of treatment with 6-HMDN; (**C**) western blot analysis showing the effects of 6-HMDN on the expression of caspase 9, caspase 3, Bcl-2, Bax, and cleaved caspase 3; (**D**) histogram of the relative protein expression levels. Data are presented as the mean ± SD. ** *p* < 0.01 and *** *p* < 0.001 compared with the control group, n = 3. Original western blots can be found in supplementary materials.

The in vitro and in vivo anti-tumor activities of 6-HMDN prompted us to investigate its underlying mechanism of action, focusing on key anti-tumor pathways. To determine whether the observed cytotoxicity of 6-HMDN was mediated by apoptosis induction, we conducted flow-cytometry-based apoptosis assays. As shown in Figure 3B, treatment with 6-HMDN resulted in dose-dependent increased in the proportions of early- and late-apoptotic cells compared with the control group. After 48 h of treatment, the percentage of apoptotic cells increased from 12.53% (control) to 74.00% (5 μM), 81.97% (10 μM), and 89.57% (20 μM). These findings demonstrate that 6-HMDN significantly induces apoptosis in HepG2 cells.

Cell apoptosis is a complex process involving multiple signaling pathways [26], including caspase activation, alterations in mitochondrial membrane permeability, modulation of the balance between pro-apoptotic and anti-apoptotic proteins, and upregulation of p53 expression [27]. The Bcl-2 protein family comprises pro-apoptotic members (Bak and Bax) and anti-apoptotic proteins (Bcl-2 and Bcl-xL). Bax, a pro-apoptotic member of the Bcl-2 family and a p53 target, can, upon increased expression, facilitate cytochrome c release from mitochondria into the cytoplasm, thereby activating downstream apoptotic signaling cascades [28]. As shown in Figure 3D, 6-HMDN treatment led to a significant increase in Bax protein expression at a 20 μM concentration, while the expression levels of Bcl-2, caspase-3, and caspase-9 proteins were significantly decreased. Concurrently, cleaved caspase-3 protein expression was notably upregulated. These results suggest that 6-HMDN-induced Bax upregulation disrupts the homeostatic balance between pro- and anti-apoptotic proteins within the Bcl-2 family, promoting cytochrome c release from mitochondria, potentially leading to the activation of the caspase-dependent apoptotic signaling pathway. The observed decrease in Bcl-2, caspase-3, and caspase-9 protein expression, coupled with cleaved caspase-3 upregulation, further supports this hypothesis. Thus, the mechanism by which 6-HMDN induces apoptosis likely involves the Bax/Bcl-2 pathway, which may play a critical role in the anti-tumor activity of 6-HMDN.

### 3.5. 6-HMDN Increased Intracellular ROS Levels in HepG2 Cells

**Figure 4:**
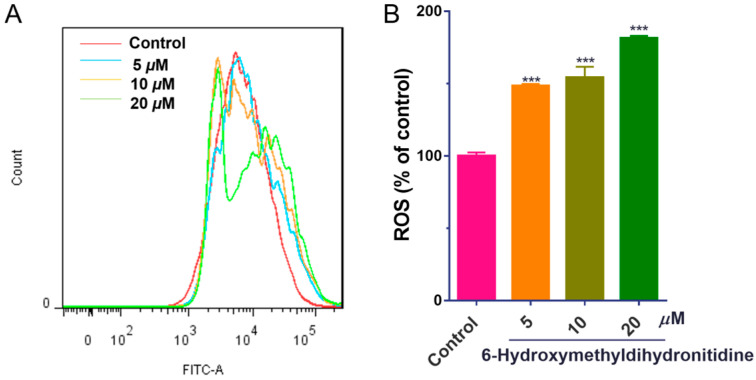
6-HMDN stimulated ROS production in HepG2 cells: (**A**) HepG2 cells were treated with different concentrations of 6-HMDN for 48 h, stained with DCFH-DA, and analyzed by flow cytometry; (**B**) histogram of relative ROS levels compared with the control group. Data are presented as the mean ± SD. *** *p* < 0.001 compared with the control group, n = 3.

Oxidative stress plays a significant role in tumorigenesis and cancer progression. Elevated ROS levels can exert anti-tumor effects through mechanisms, such as ferroptosis induction, apoptosis triggering, and cell cycle regulation [29]. To investigate whether 6-HMDN exerts its anti-tumor effect via oxidative stress mechanisms, we utilized the DCFH-DA fluorescent probe and flow cytometry to measure ROS levels in HepG2 cells. Experimental data (Figure 4) revealed that 48 h of treatment with 6-HMDN significantly increased intracellular ROS levels in HepG2 cells in a dose-dependent manner. Compared with the control group, the ROS levels increased by 0.5-fold in the 5 μM 6-HMDN treatment group, 0.6-fold in the 10 μM group, and 0.8-fold in the 20 μM group. These results suggest that 6-HMDN-induced ROS elevation may synergistically inhibit tumor cell proliferation through a multi-faceted redox regulatory network.

### 3.6. 6-HMDN Activated Ferroptosis

**Figure 5:**
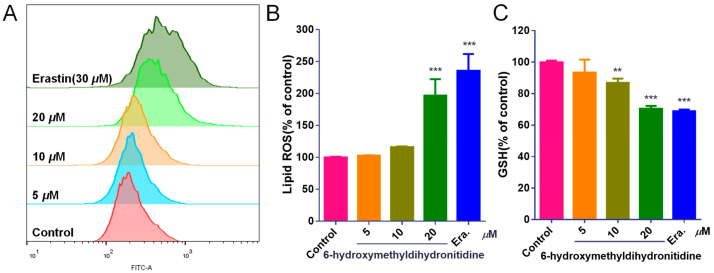
6-HMDN activated ferroptosis in HepG2 cells: (**A**) HepG2 cells were treated with different concentrations of 6-HMDN for 24 h, stained with BODIPY^581/591^, and analyzed by flow cytometry; (**B**) histogram of relative lipid ROS levels compared with the control group; (**C**) HepG2 cells were treated with different concentrations of 6-HMDN for 24 h. Histogram of relative GSH levels compared with the control group. Erastin (30 μM) was used as a positive control for ferroptosis induction. Data are presented as the mean ± SD. ** *p* < 0.01 and *** *p* < 0.001 compared with the control group, n = 3.

Ferroptosis, an iron-dependent form of regulated cell death characterized by lipid peroxidation, has emerged as a promising target for cancer therapy [30]. Glutathione peroxidase 4 (GPX4) is a key enzyme in the cellular antioxidant system, primarily responsible for catalyzing the reduction in lipid hydroperoxides using glutathione (GSH) as a cofactor [31]. To determine whether 6-HMDN exerts its anti-tumor effect through the ferroptosis pathway, we assessed lipid ROS levels and GSH content in HepG2 cells following drug treatment. The BODIPY^581/591^ fluorescent probe was used to measure lipid peroxidation. Flow cytometry analysis (Figure 5B) demonstrated a dose-dependent increase in lipid ROS levels in 6-HMDN-treated cells. At a 6-HMDN concentration of 20 μM, lipid ROS levels increased by 96.47%. Concurrently, GSH assays (Figure 5C) revealed a significant dose-dependent decrease in intracellular GSH concentration following 24 h of 6-HMDN treatment. GSH levels decreased by 6.57% in the 5 μM group, 13.22% in the 10 μM group, and 29.58% in the 20 μM group compared to the control group. Notably, the dose-dependent GSH depletion was inversely correlated with the dose-dependent increase in lipid ROS, a phenomenon consistent with the hallmarks of ferroptosis. This suggests that 6-HMDN may trigger a lipid peroxidation cascade by disrupting the GSH-GPX4 antioxidant axis, ultimately initiating ferroptotic cell death. These findings provide direct biochemical evidence for the activation of ferroptosis by 6-HMDN.

### 3.7. 6-HMDN Reduced HepG2 Cell Migration

**Figure 6:**
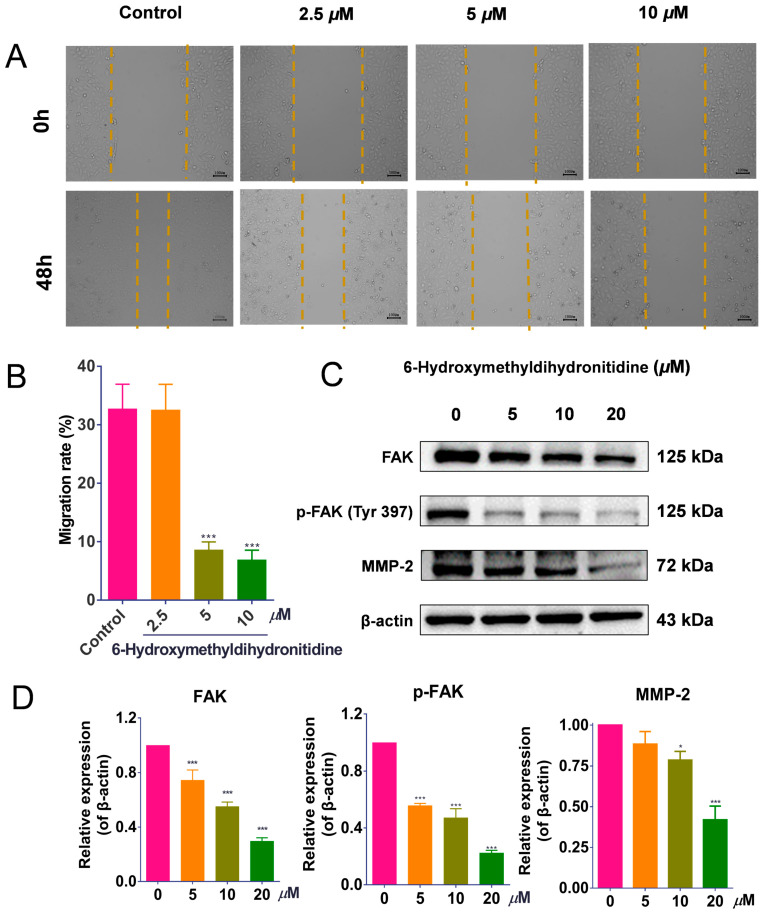
6-HMDN inhibited HepG2 cell migration by regulating the FAK/MMP2 signaling pathway: (**A**) representative images of HepG2 cell morphology at 0 and 48 h after 6-HMDN treatment; (**B**) quantitative analysis of the cell migration rate (%) using ImageJ software; (**C**) Western blot analysis of FAK, p-FAK, and MMP-2 expression in 6-HMDN-treated cells; (**D**) histogram of the relative protein expression levels. Data are presented as the mean ± SD. * *p* < 0.05 and *** *p* < 0.001 compared with the control group, n = 3. Original western blots can be found in supplementary materials.

To investigate the effect of 6-HMDN on HepG2 cell migration, we performed a wound-healing assay. Following 48 h of treatment with 6-HMDN, the migration rate of HepG2 cells decreased from 32.72% (control) to 8.60% (5 μM) and 6.90% (10 μM) (Figure 6B). These results indicate that 6-HMDN significantly inhibited HepG2 cell migration in vitro.

Activation of FAK has been shown to induce the expression of matrix metalloproteinases (MMPs), which further promotes cancer cell invasiveness [32]. As shown in Figure 6D, 48 h of treatment of tumor cells with 6-HMDN significantly inhibited both total FAK protein levels and FAK phosphorylation. Consistent with this, MMP-2 protein expression was downregulated in a concentration-dependent manner. These findings suggest that 6-HMDN may inhibit HepG2 cell migration by modulating the FAK/MMP signaling pathway.

## 4. Discussion

The current study demonstrates the significant anti-tumor activity of 6-HMDN, with its mechanism of action encompassing multiple aspects, including the inhibition of tumor cell proliferation and migration, as well as the promotion of ferroptosis.

**Scheme 1:**
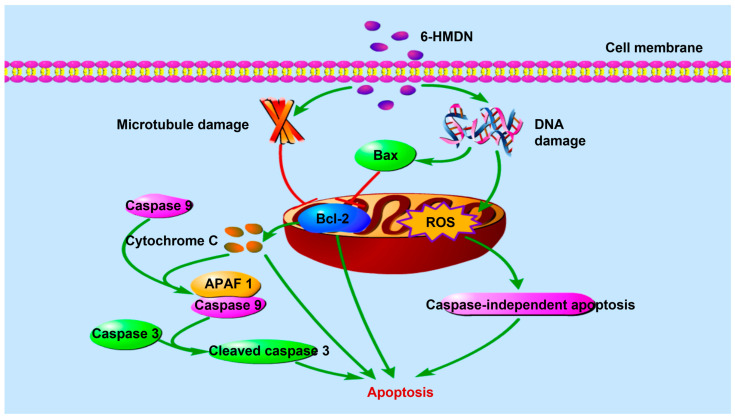
Signaling pathway of 6-HMDN-induced tumor cell apoptosis.

Regarding the inhibition of tumor cell proliferation, our results indicate that 6-HMDN potently induces apoptosis in HepG2 cells, with the proportion of apoptotic cells increasing in a dose-dependent manner. Further mechanistic investigation revealed that 6-HMDN may disrupt the balance between pro- and anti-apoptotic proteins by modulating the Bax/Bcl-2 pathway, as shown in Scheme 1. This, in turn, promotes cytochrome c release and the subsequent activation of the caspase-dependent apoptosis signaling cascade [33]. By comparison, it can be found that the apoptosis mechanism of 6-HMDN partially overlaps with classical alkaloids such as colchicine [34] and camptothecin [35]. However, the MTT results and SI show that 6-HMDN exhibited a stronger effect on inducing the specific apoptosis of liver cancer cells. It can be reasonably inferred that 6-HMDN not only inherits the advantages of some alkaloids in regulating mitochondrial pathways to induce apoptosis but may also have unique targets or regulatory mechanisms, which can be further explored in the future.

**Scheme 2:**
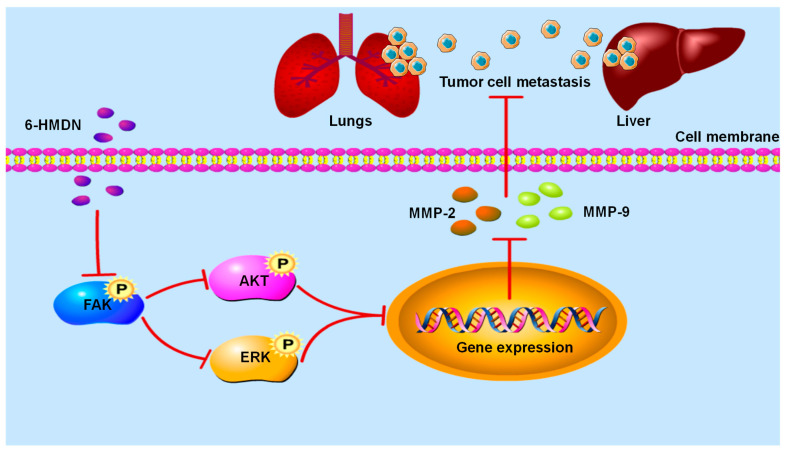
Signaling pathway of 6-HMDN-mediated inhibition of tumor cell migration.

Tumor cell metastasis is the main determinant of cancer mortality. This study found that 6-HMDN significantly inhibited the migration ability of HepG2 cells. Compared with other classic alkaloids, such as berberine [36] and indirubin [37], which are known to inhibit the migration of tumor cells in vitro by affecting FAK and MMP, 6-HMDN also acts on the FAK/MMP signaling pathway to achieve its anti-migration effect. Specifically, as shown in Scheme 2, 6-HMDN inhibits the phosphorylation of FAK, thereby downregulating the expression of MMP-2, while FAK activation typically promotes the expression of MMP to enhance the invasiveness of cancer cells [38]. Our results indicate that 6-HMDN inhibits FAK phosphorylation and downregulates MMP-2 expression, leading to inhibition of tumor cell migration.

**Scheme 3:**
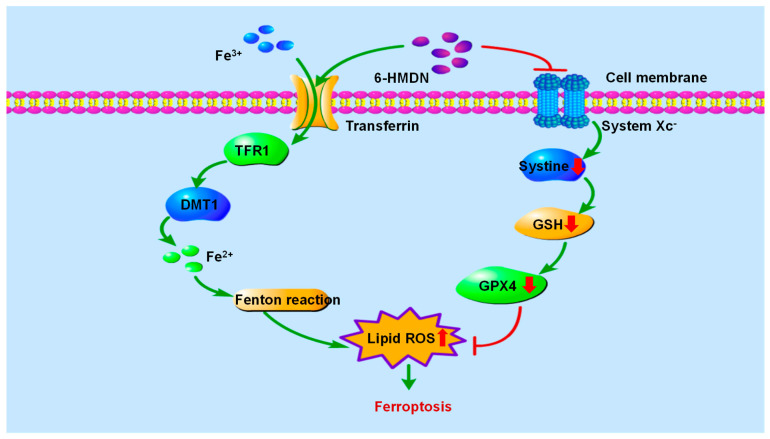
Signaling pathway of 6-HMDN-induced ferroptosis.

Iron-dependent regulation of cell death driven by lipid peroxidation has become a key therapeutic target for cancer. As shown in Scheme 3, in the mechanism of ferroptosis, GPX4 is the core regulatory factor of ferroptosis, lipid ROS is the key executive molecule, and GSH depletion is an important characteristic of ferroptosis [39]. In this study, we focused on measuring the effects of 6-HMDN on lipid ROS and glutathione levels. According to the experimental results, treating HepG2 cells with 6-HMDN resulted in a dose-dependent increase in lipid ROS levels and a decrease in GSH concentration. Existing research reports indicate that many alkaloids, such as dauricine, can directly regulate GPX4; capsaicin can inactivate the SLC7A11/GPX4 pathway and promote ferroptosis [40]. Based on existing cases of multiple alkaloids regulating ferroptosis, we reasonably speculate that 6-HMDN may trigger a lipid peroxidation cascade reaction by disrupting the GSH-GPX4 antioxidant axis, ultimately leading to ferroptosis. In this study, we focused on detecting lipid ROS levels and glutathione content, but there is still a lack of direct marker expression levels for ferroptosis, such as GPX4. Based on this preliminary exploration of 6-HMDN-activated ferroptosis, we can further explore mechanisms such as the GPX4 pathway, iron metabolism, and lipid metabolism in the future.

Furthermore, 6-HMDN significantly elevated intracellular ROS levels in tumor cells. As a critical signaling molecule, ROS can exert anti-tumor effects through various mechanisms, including ferroptosis induction [41], apoptosis initiation, and cell cycle regulation [42]. Firstly, an increase in ROS levels, especially lipid peroxides, can lead to ferroptosis by consuming GSH and subsequently inhibiting GPX4 activity. In our study, the significant increase in lipid ROS levels and dose-dependent depletion of GSH after 6-HMDN treatment strongly indicate the activation of ferroptosis. Secondly, excessive ROS can lead to mitochondrial dysfunction and activate the caspase-dependent apoptotic cascade. The changes in the expression levels of apoptosis-related proteins after treatment with 6-HMDN may indicate that ROS may have activated apoptosis. Specifically, the relationship between ROS, ferroptosis, and apoptosis is not mutually exclusive but rather synergistic. Elevated ROS can trigger ferroptosis, and as cell damage accumulates due to long-term oxidative stress, apoptosis mechanisms may be reactivated to ensure cell elimination. The interaction between these two cell death modes can enhance the anti-tumor efficacy of 6-HMDN. Based on the literature review and experimental results, it is believed that 6-HMDN may synergistically inhibit tumor cell proliferation through a multidimensional redox regulatory network.

## 5. Conclusions

This study systematically evaluated the anti-tumor activity and mechanisms of action of 6-HMDN, a natural alkaloid isolated from *Z. ailanthoides*. Utilizing in vitro cell-based assays and in vivo zebrafish models, the anti-tumor effects and underlying mechanisms of 6-HMDN were explored. Our findings demonstrate that 6-HMDN effectively inhibited the proliferation of HepG2 and MCF-7 cells in vitro and significantly suppressed the migration of HepG2 cells. Mechanistic studies revealed that its anti-tumor effects involved the regulation of multiple pathways, as follows: induction of apoptosis through the modulation of apoptosis-related proteins (e.g., Bcl-2/Bax) and activation of the caspase cascade; triggering ferroptosis by increasing intracellular ROS and lipid ROS levels while reducing GSH content; and attenuating cell migration by inhibiting FAK phosphorylation. Importantly, in vivo experiments using a zebrafish xenograft model further confirmed that 6-HMDN effectively inhibited tumor cell proliferation and metastasis. In conclusion, as a natural product exhibiting significant anti-tumor activity both in vitro and in vivo, 6-HMDN exerts its effects through the coordinated regulation of signaling pathways associated with apoptosis, ferroptosis, and migration, and it holds considerable promise as a novel multi-targeted anti-cancer drug candidate.

## Figures and Tables

**Table 1:** ^13^C NMR and ^1^H NMR data of 6-HMDN in CDCl_3_ (*δ* in ppm and *J* in Hz).

Position	*δ*_C_	*δ*_H_
1	104.7	7.13, s
2	147.6	
3	148.5	
4	99.8	7.65, s
4a	127.0	
4b	137.9	
6	65.4	4.15, dd (10.7, 3.8)
6a	124.0	
7	110.7	6.80, s
8	149.1	
9	149.1	
10	106.4	7.34, s
10a	124.3	
10b	123.5	
11	119.6	7.69, d (8.5)
12	124.3	7.52, d (8.5)
12a	131.0	
13	101.2	6.06, s
14	62.9	3.15, t (10.7) 3.47, dd (10.7, 3.8)
8-OMe	56.1	4.00, s
9-OMe	56.1	3.96, s
*N*-Me	42.8	2.74, s

## Data Availability

Data are contained within the article and Supplementary Materials.

## Supplementary Materials

The following are available online at https://www.mdpi.com/article/10.3390/biom15060814/s1, Figure S1: Toxic effects of 6-HMDN on zebrafish embryos; Figure S2: ^1^H NMR spectrum of 6-HMDN; Figure S3: ^13^C NMR spectrum of 6-HMDN; Figure S4: DEPT spectrum of 6-HMDN; File S1: The original western blot images.
