# Supplementary material for: A Natural Alkaloid, 6-Hydroxymethyldihydronitidine, Suppresses Tumor Progression by Co-Regulating Apoptosis, Ferroptosis, and FAK Pathways
Source: Biomolecules. 2025 Jun 4;15(6):814. doi: 10.3390/biom15060814 (PMC12190358; doi:10.3390/biom15060814)
Supplement: Supplementary file 1 [file biomolecules-15-00814-s001.zip › File S1 The original western blot images.pdf]

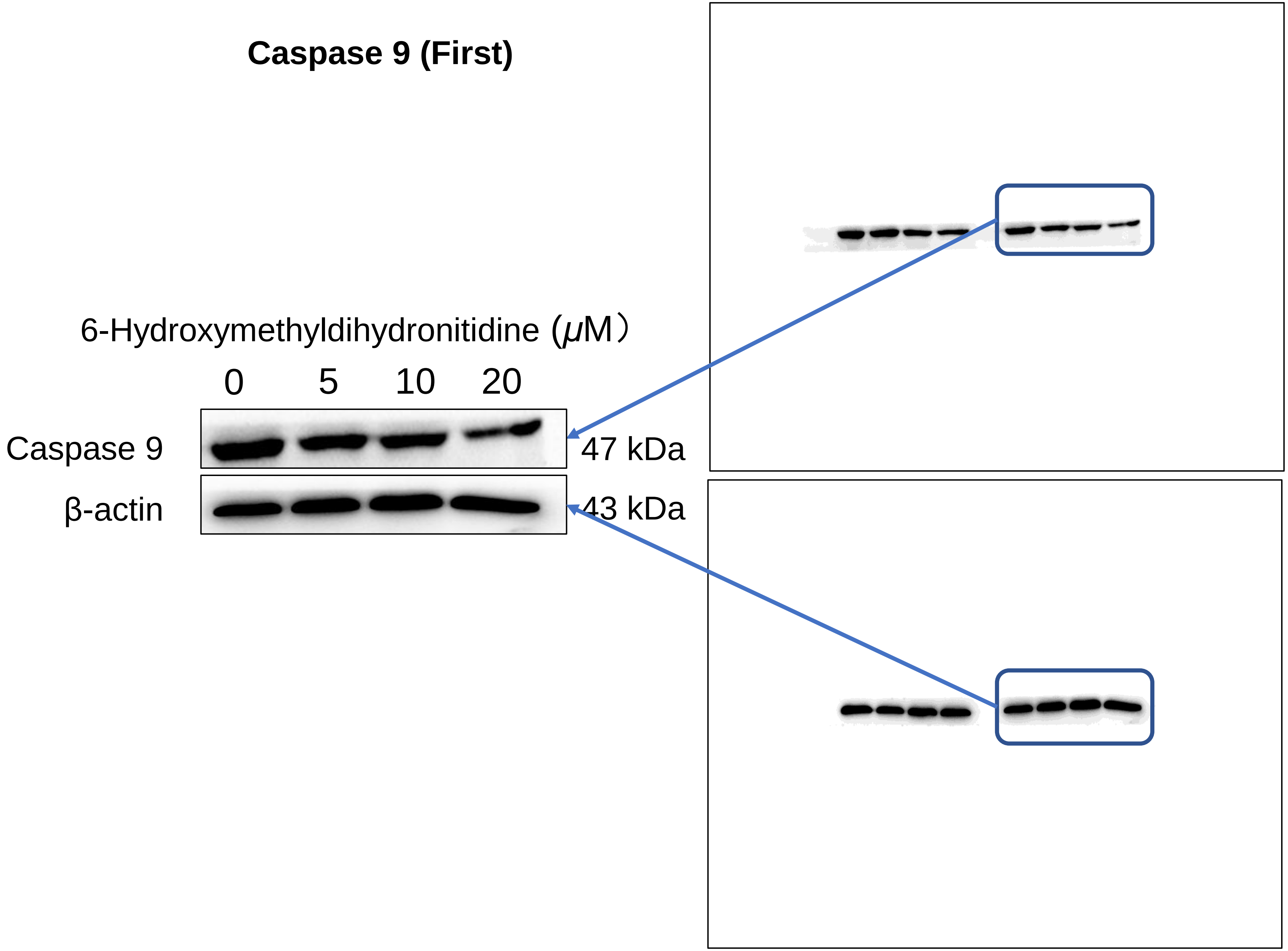
Caspase 9 (First)
6-Hydroxymethyldihydronitidine (μM)
0
5
10
20
Caspase 9
β-actin
47 kDa
43 kDa

Caspase 9 (Second)

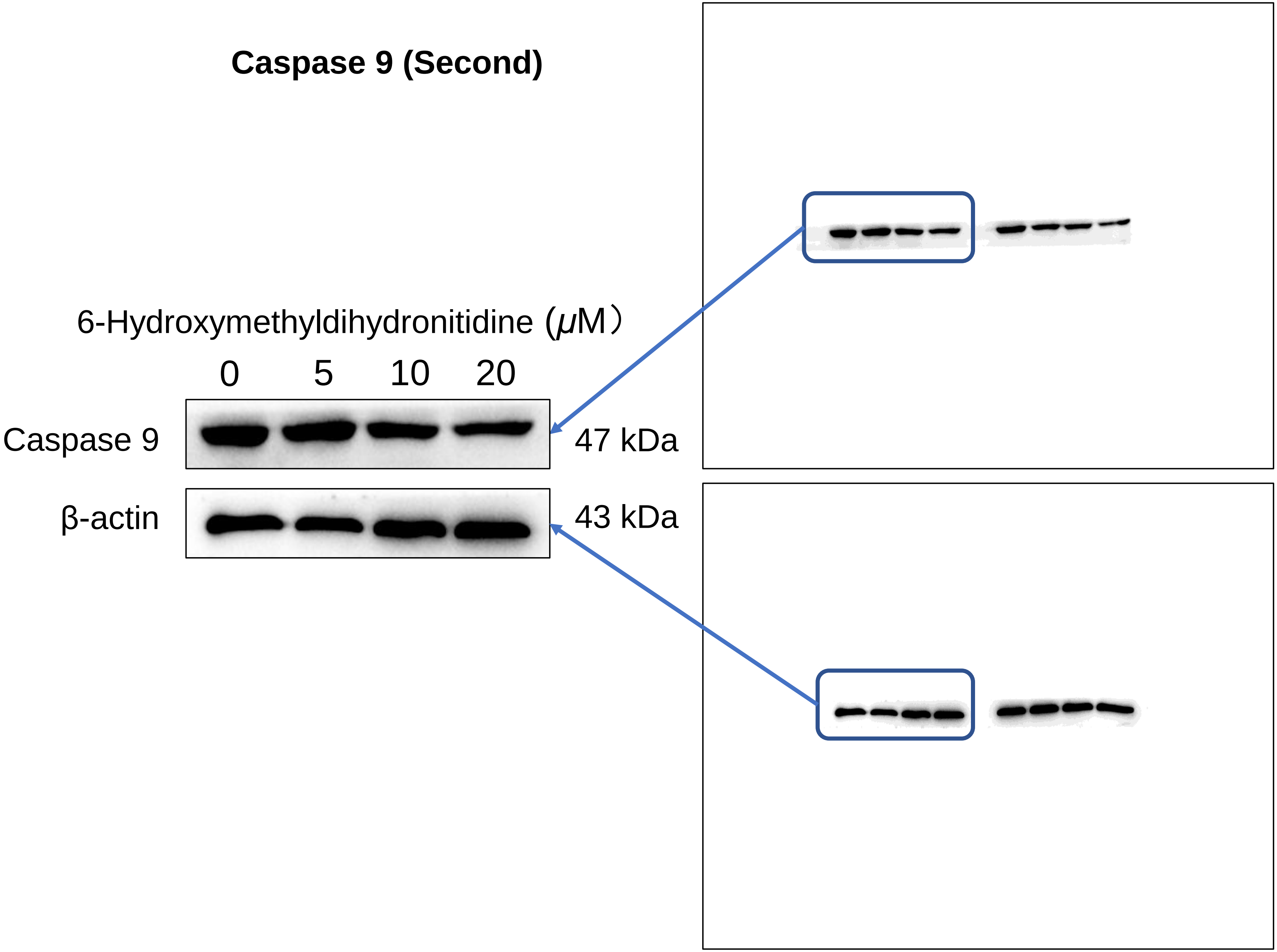

Caspase 9 (Third)

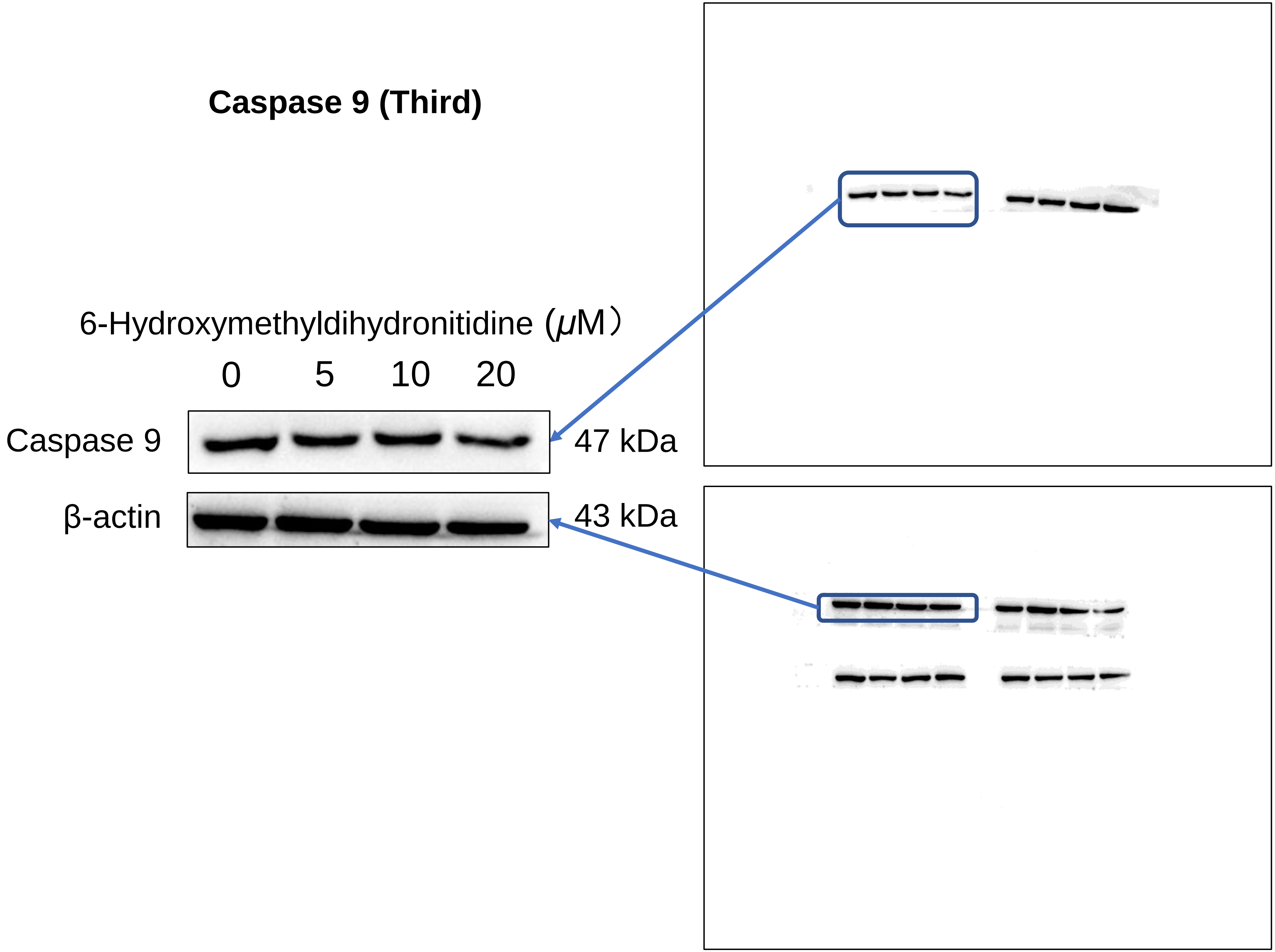

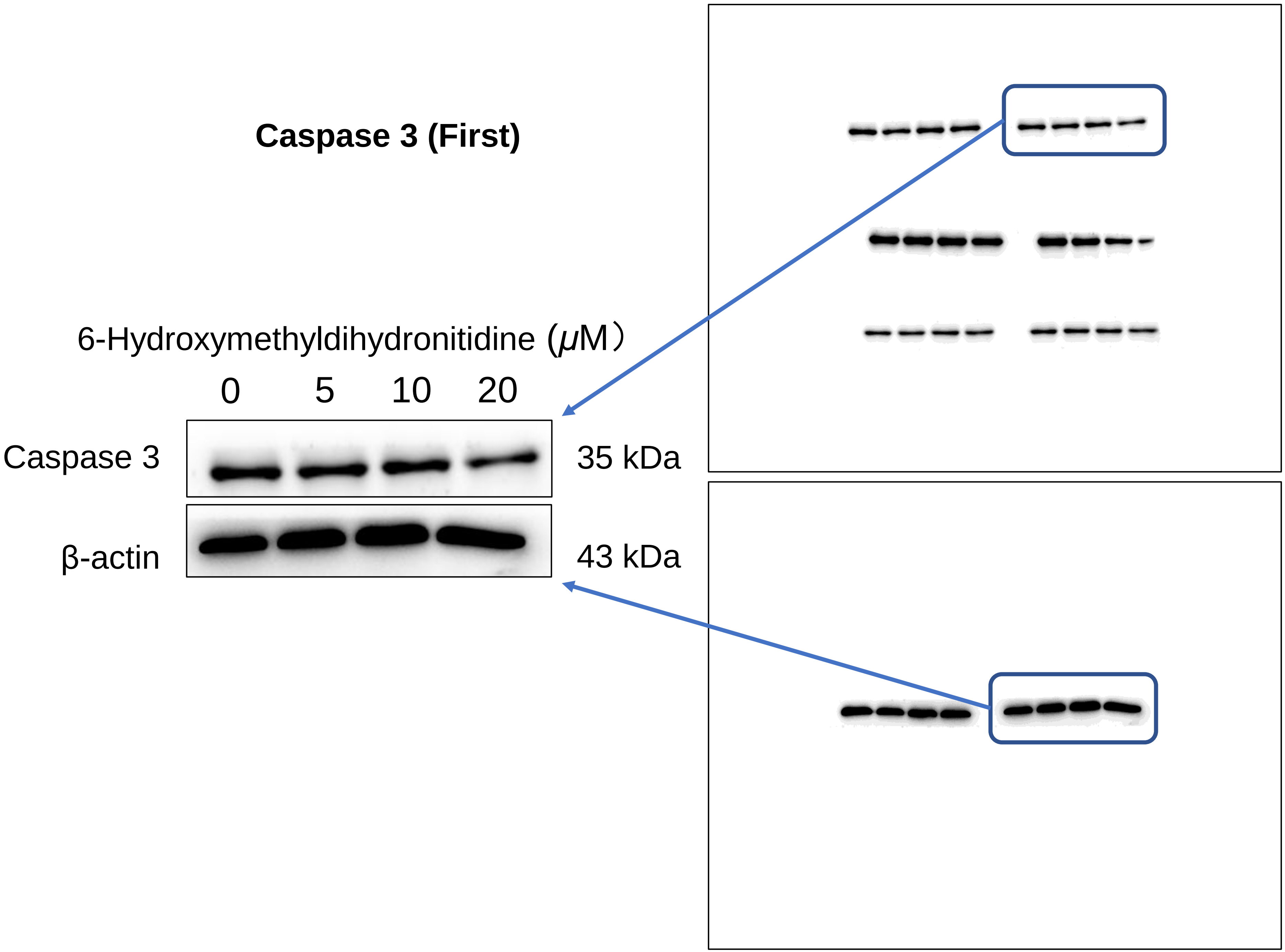
Caspase 3 (First)
6-Hydroxymethyldihydronitidine (μM)
0
5
10
20
Caspase 3
35 kDa
β-actin
43 kDa

Caspase 3 (Second)

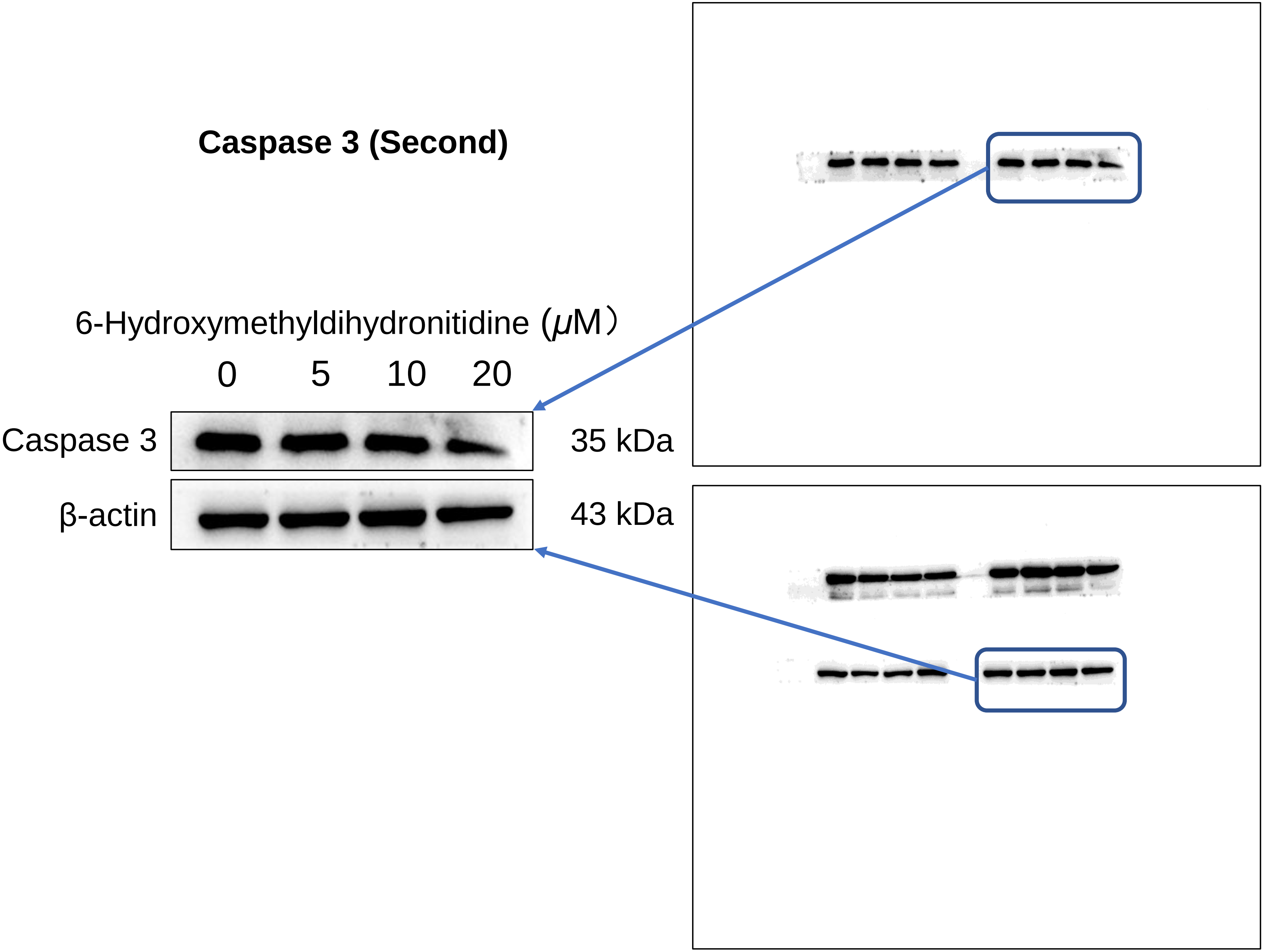

Caspase 3 (Third)

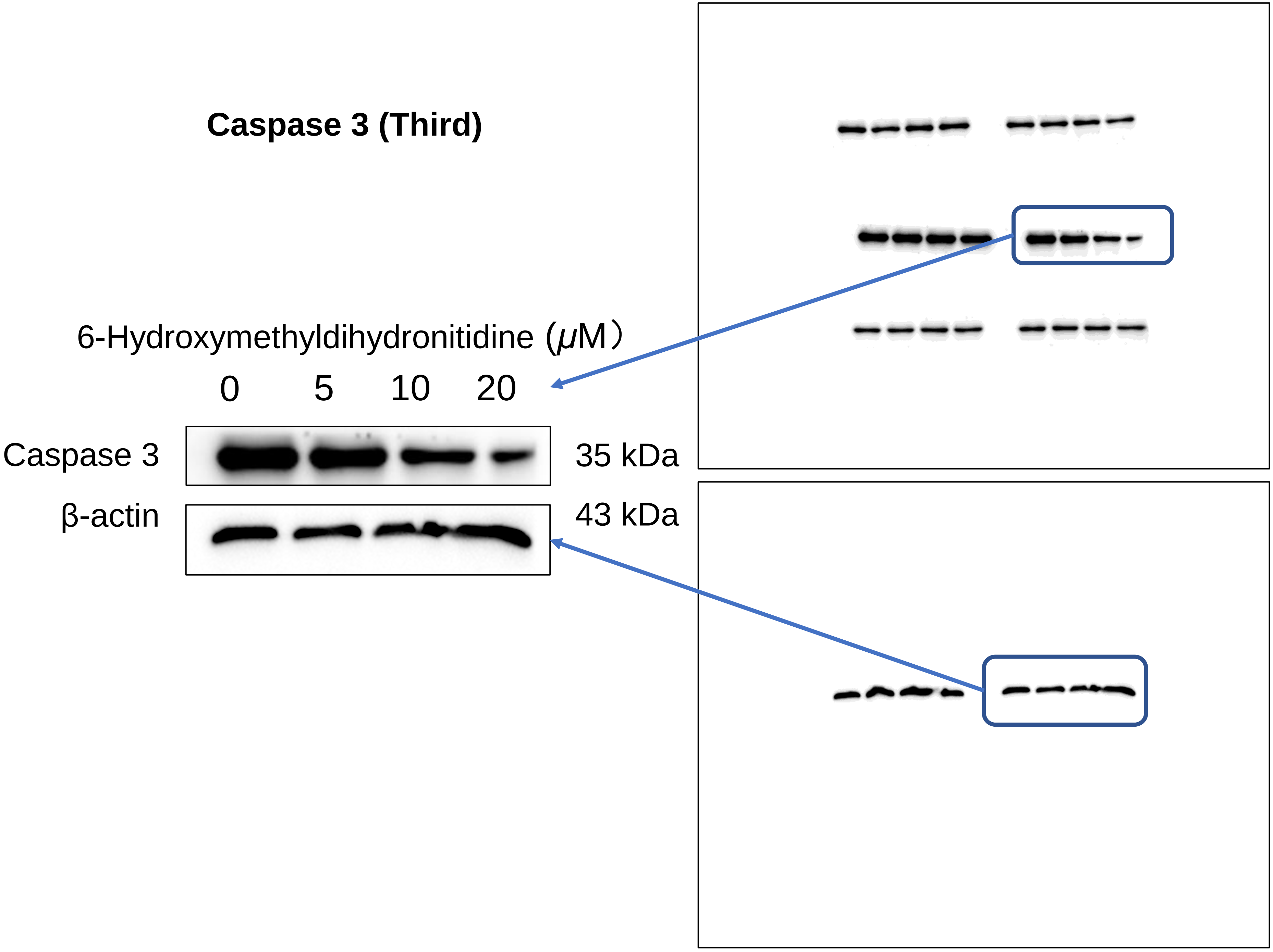

Bcl-2 (First)

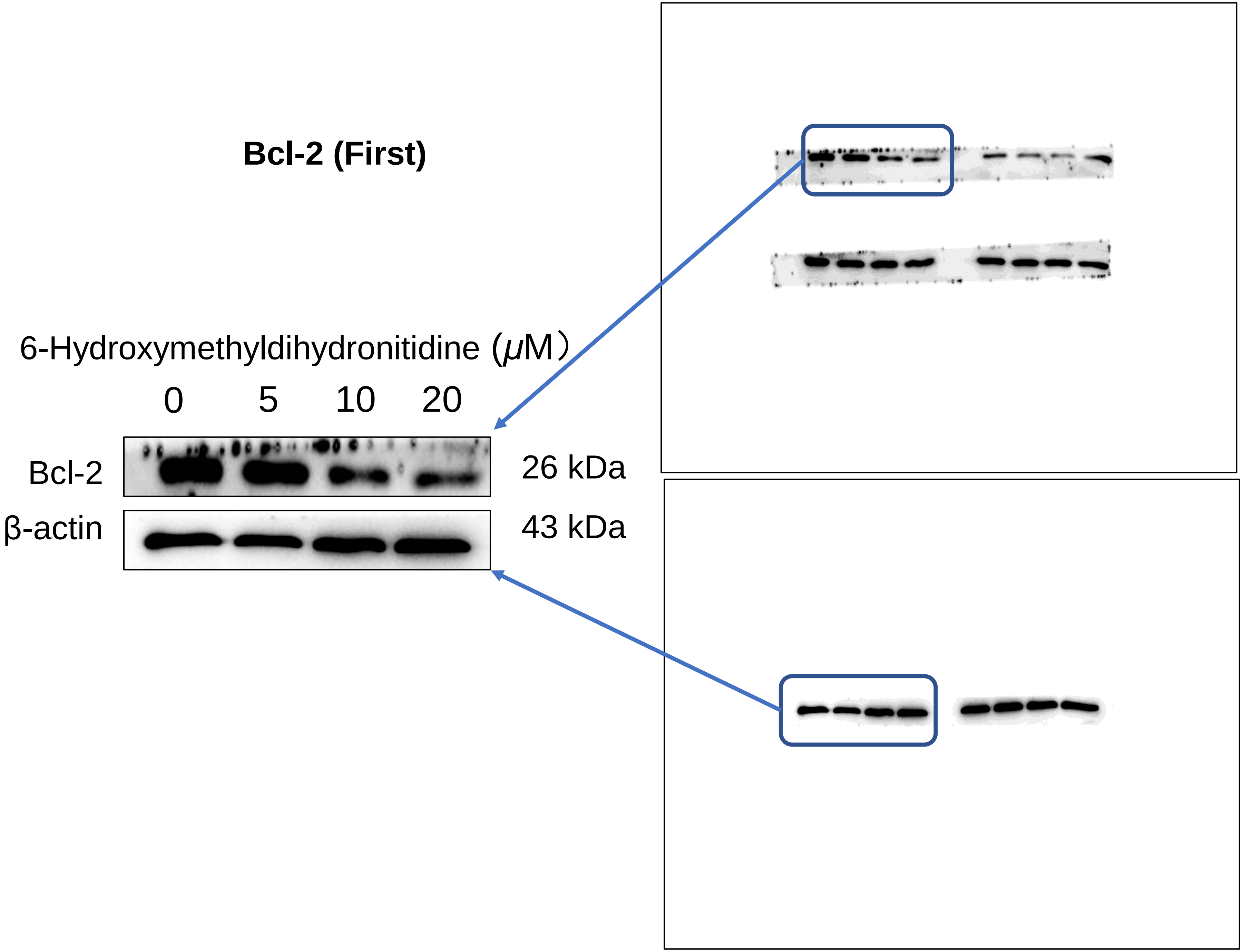
6-Hydroxymethyldihydronitidine（μM）
0
5
10
20
Bcl-2
β-actin
26 kDa
43 kDa

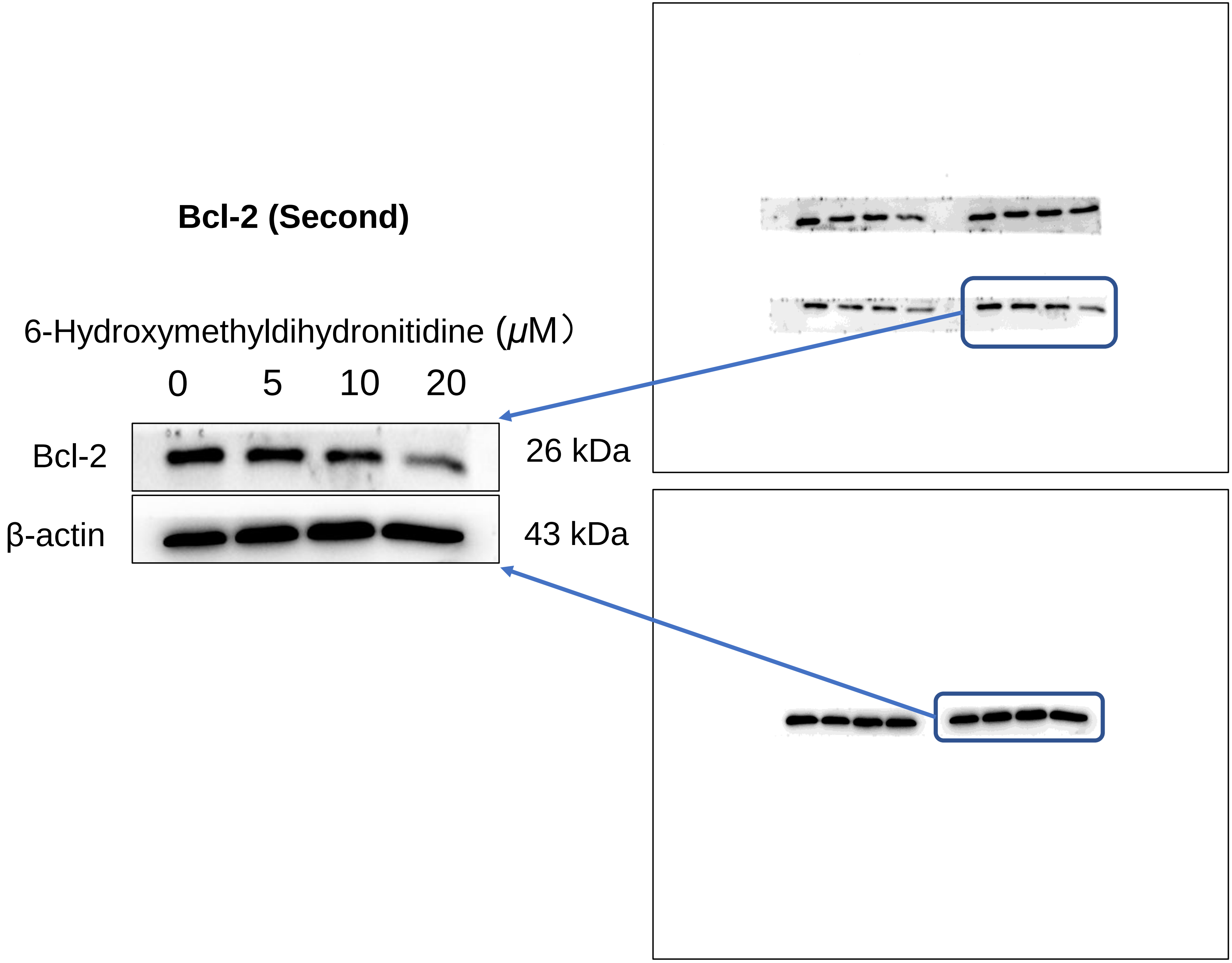

Bcl-2 (Second)
6-Hydroxymethyldihydronitidine (μM)
0
5
10
20
Bcl-2
26 kDa
β-actin
43 kDa

Bcl-2 (Third)

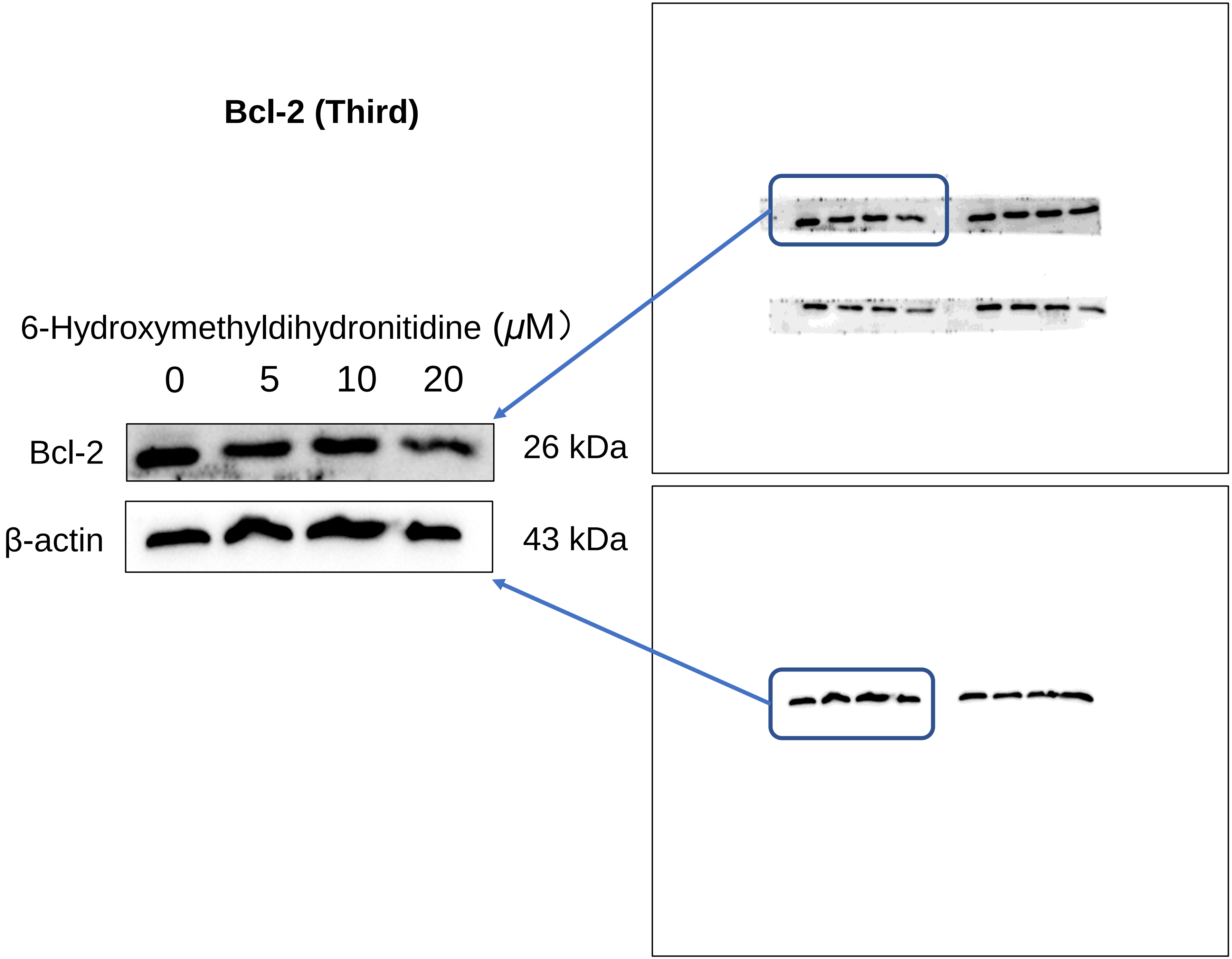

6-Hydroxymethyldihydronitidine (μM)
0
5
10
20
Bcl-2
26 kDa
β-actin
43 kDa

Bax (First)

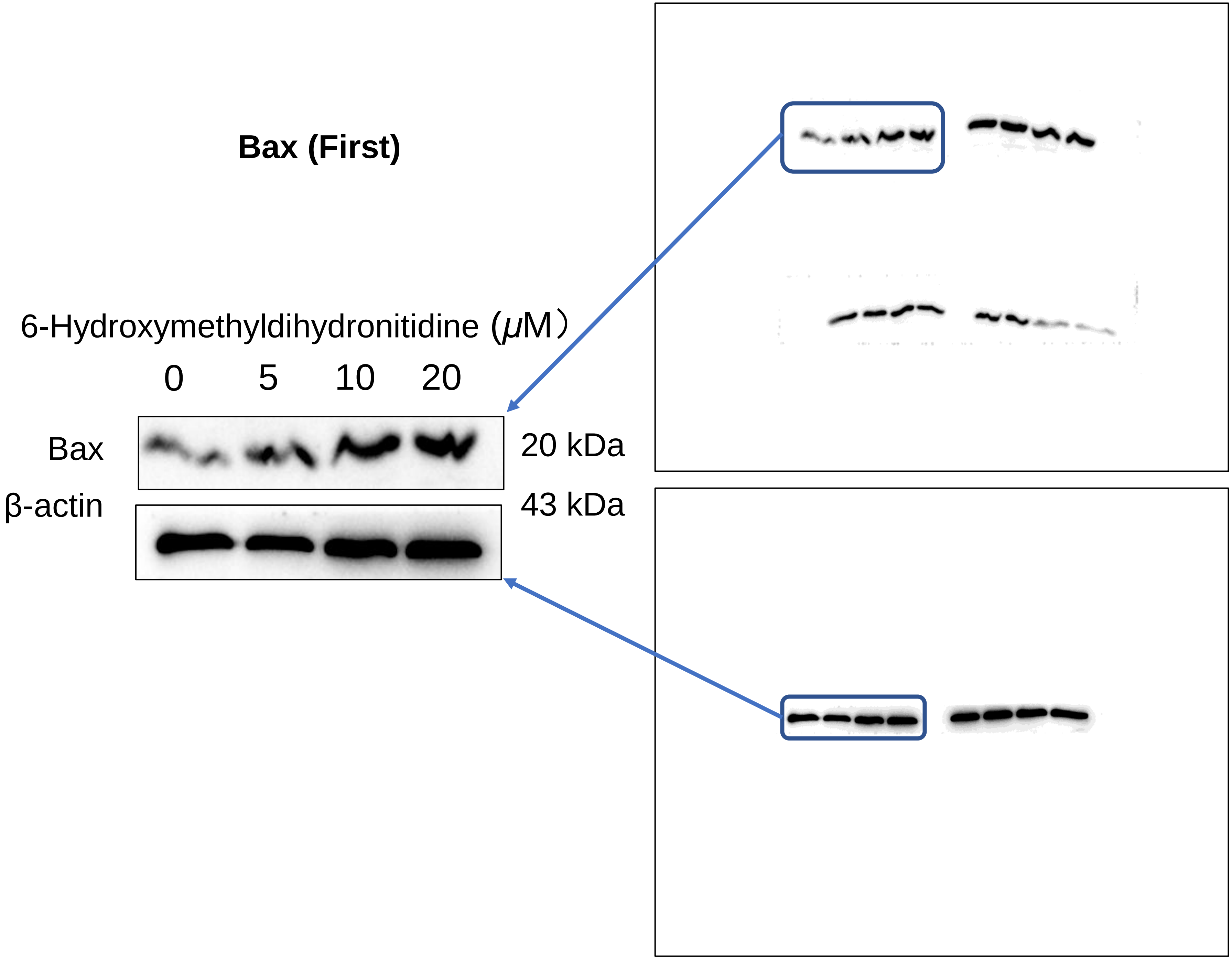
6-Hydroxymethyldihydronitidine (μM）
0
5
10
20
Bax
20 kDa
β-actin
43 kDa

Bax (Second)

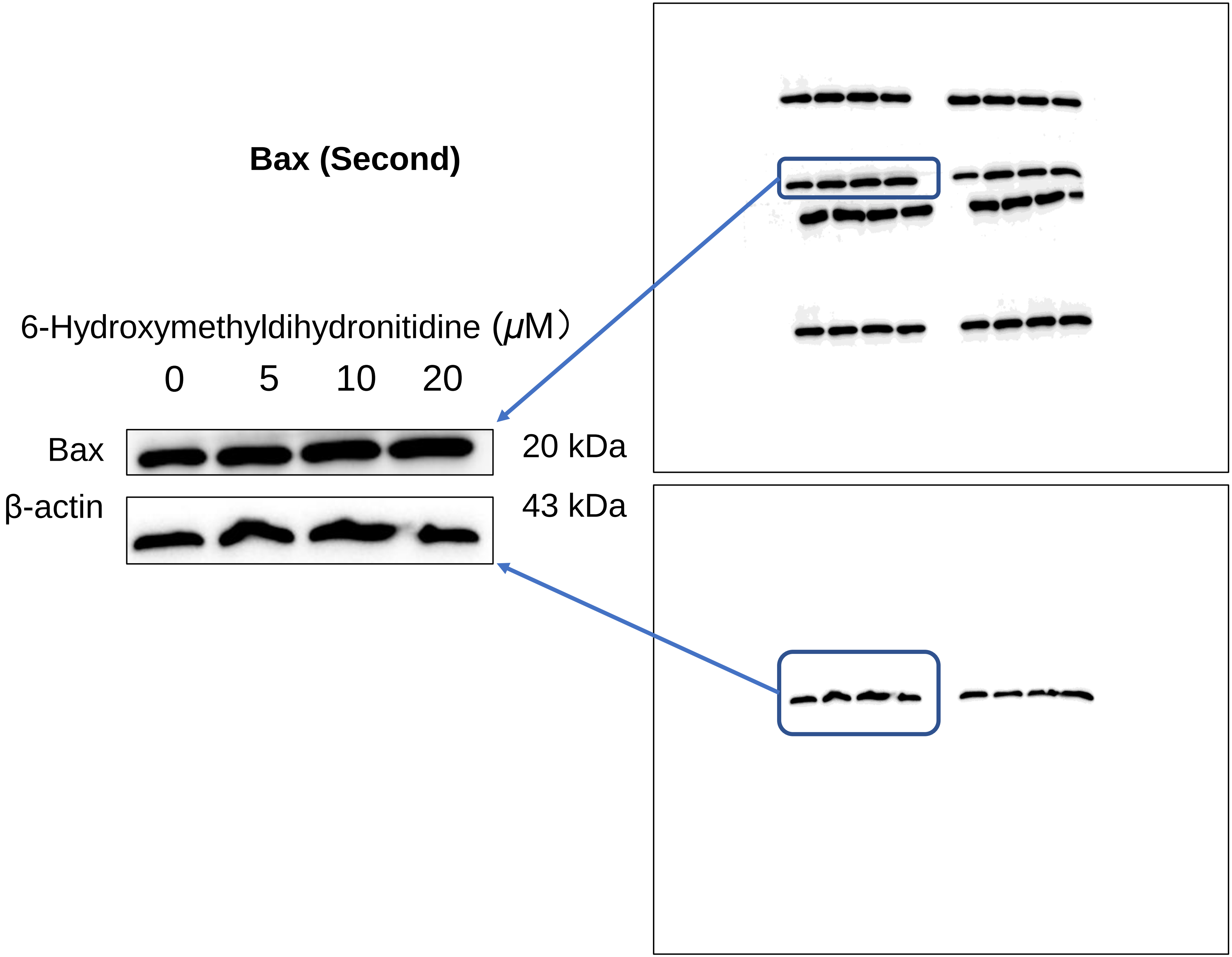
6-Hydroxymethyldihydronitidine (μM)
0
5
10
20
Bax
β-actin
20 kDa
43 kDa

Bax (Third)

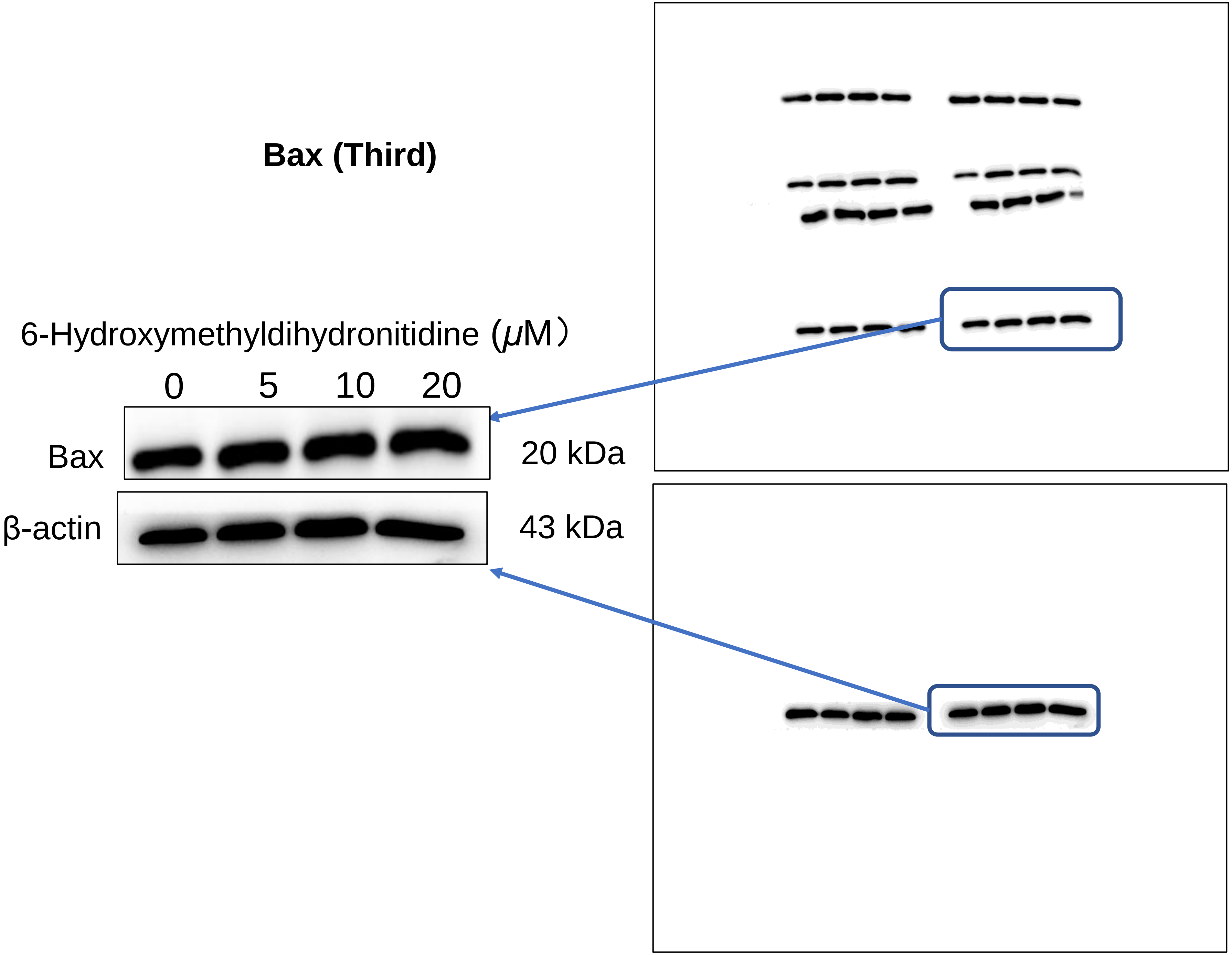
6-Hydroxymethyldihydronitidine (μM)
0
5
10
20
Bax
β-actin
20 kDa
43 kDa

Cleaved Caspase 3 (First)

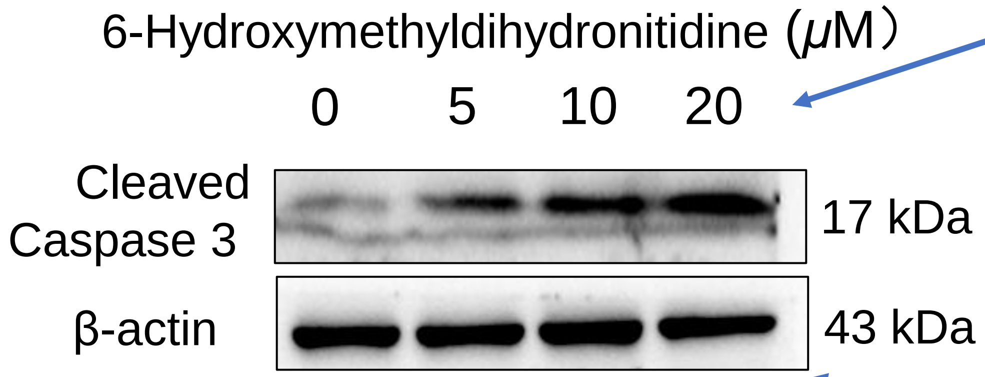

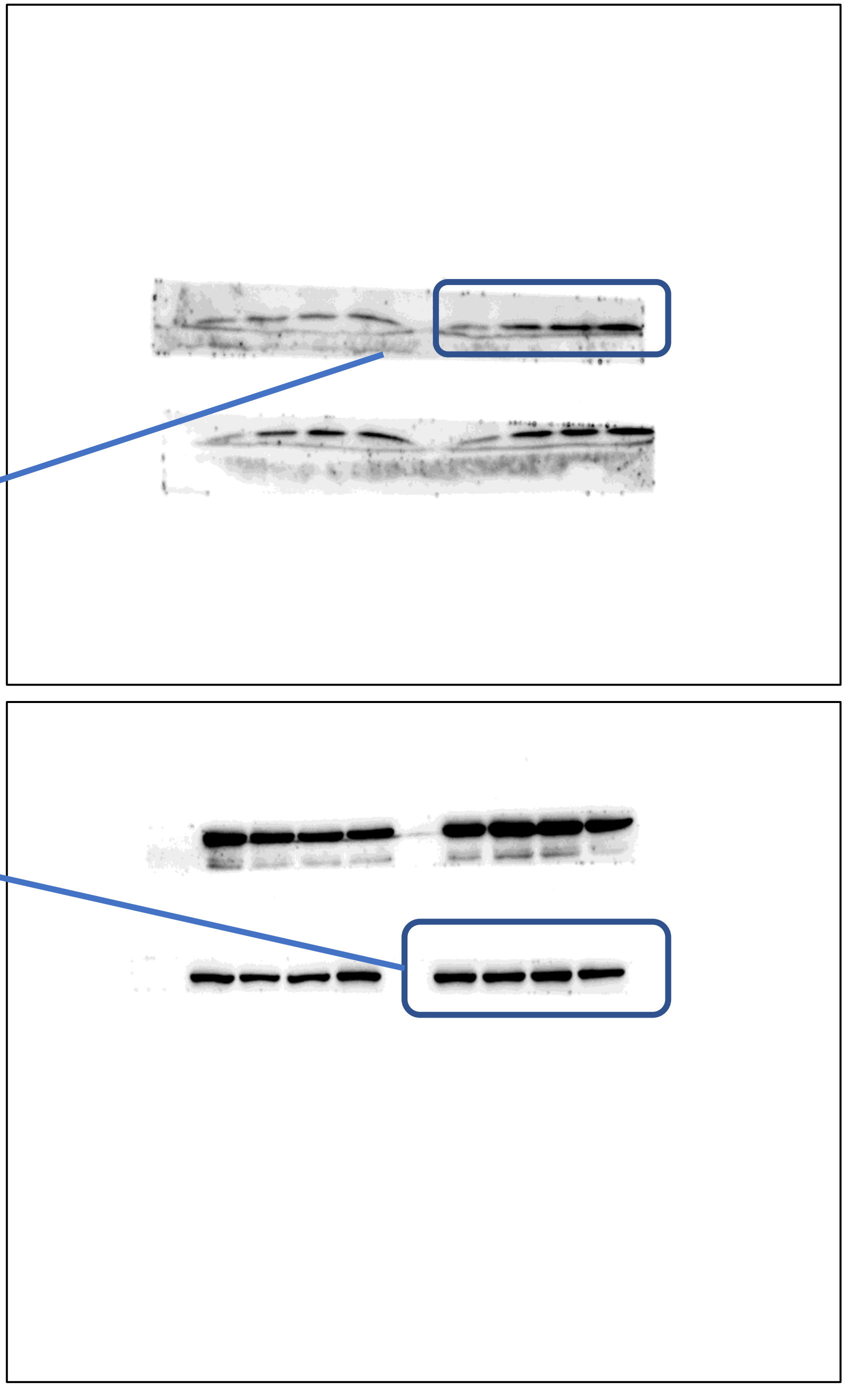

Cleaved Caspase 3 (Second)

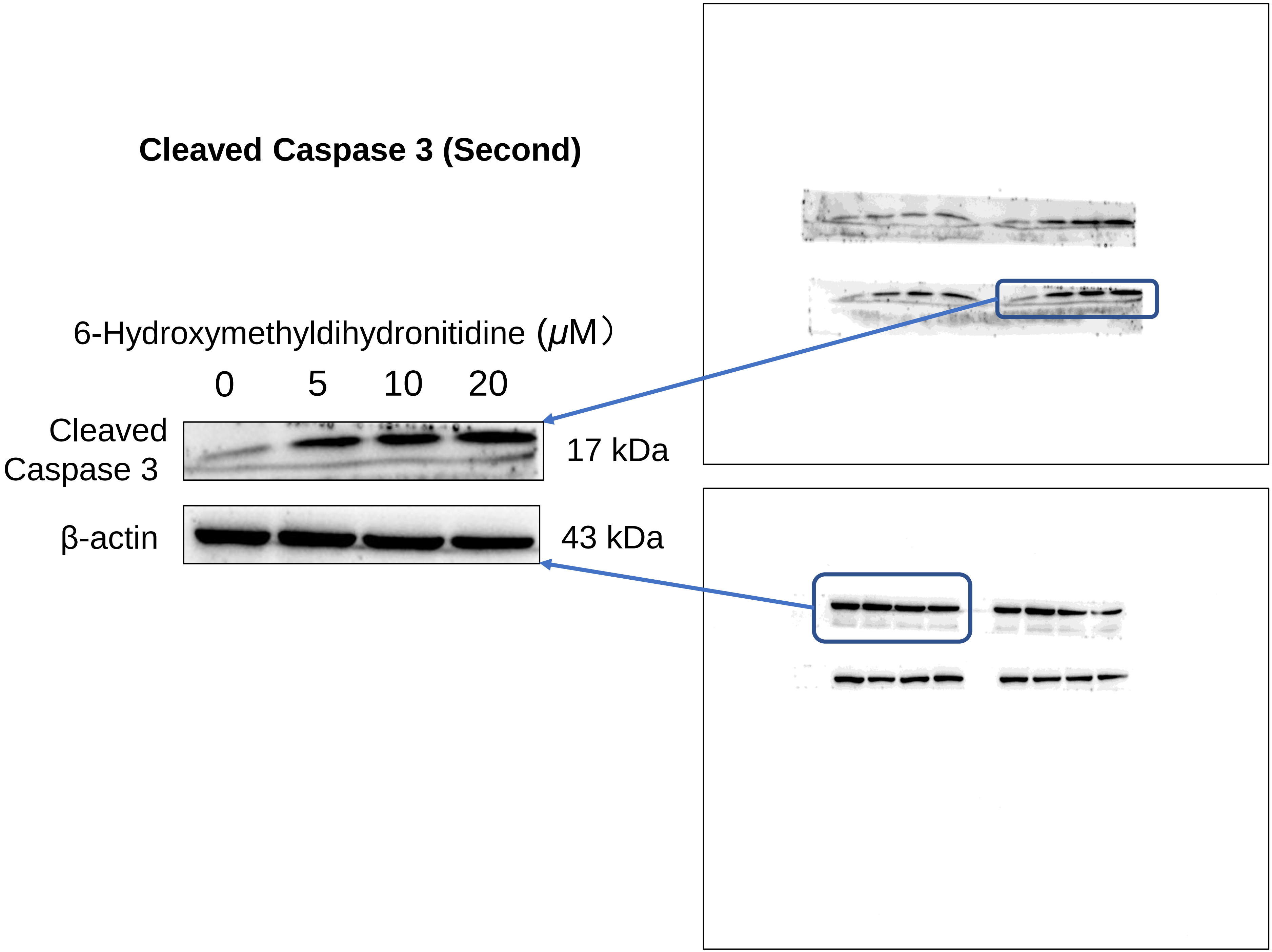

Cleaved Caspase 3 (Third)

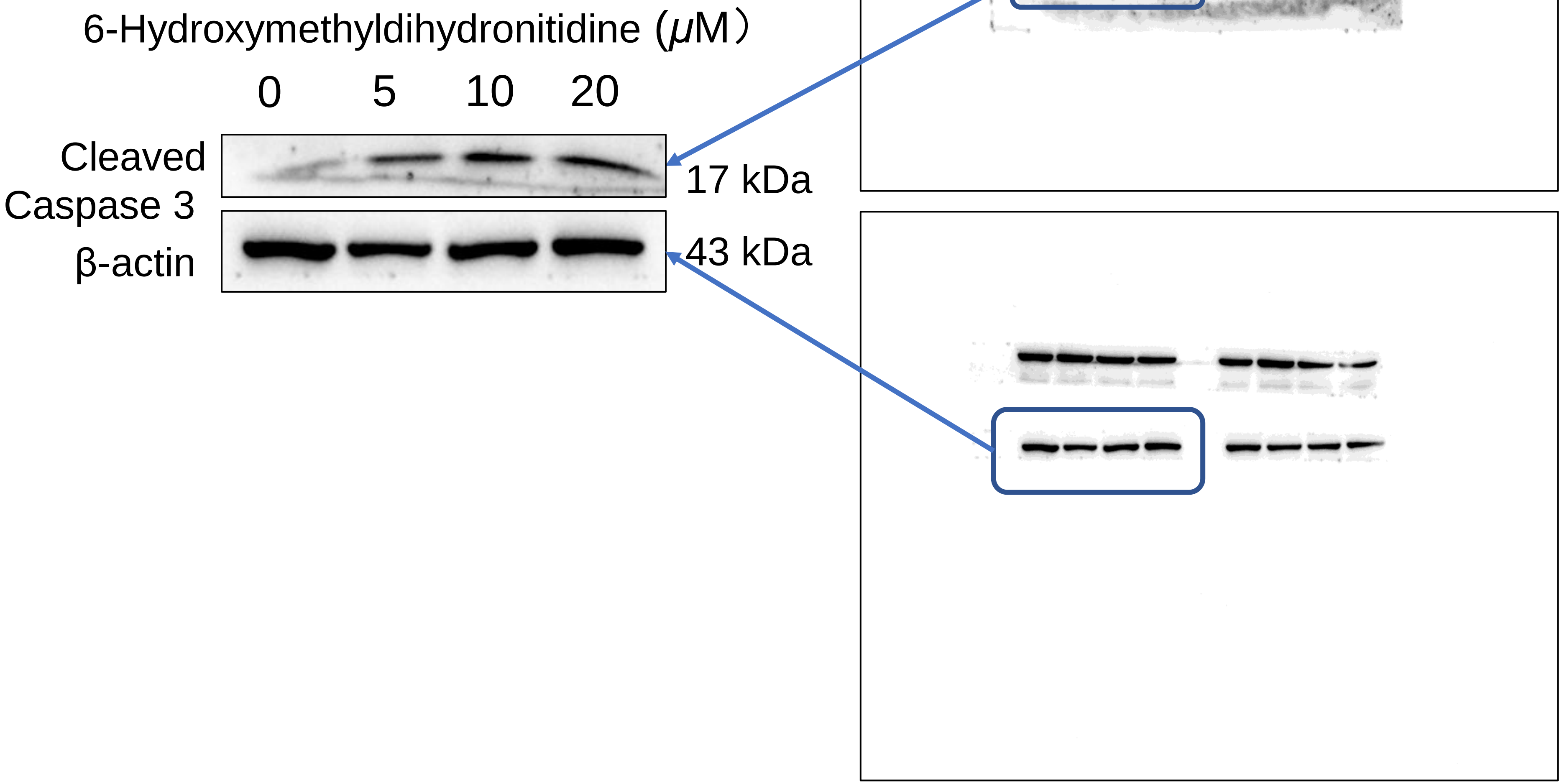

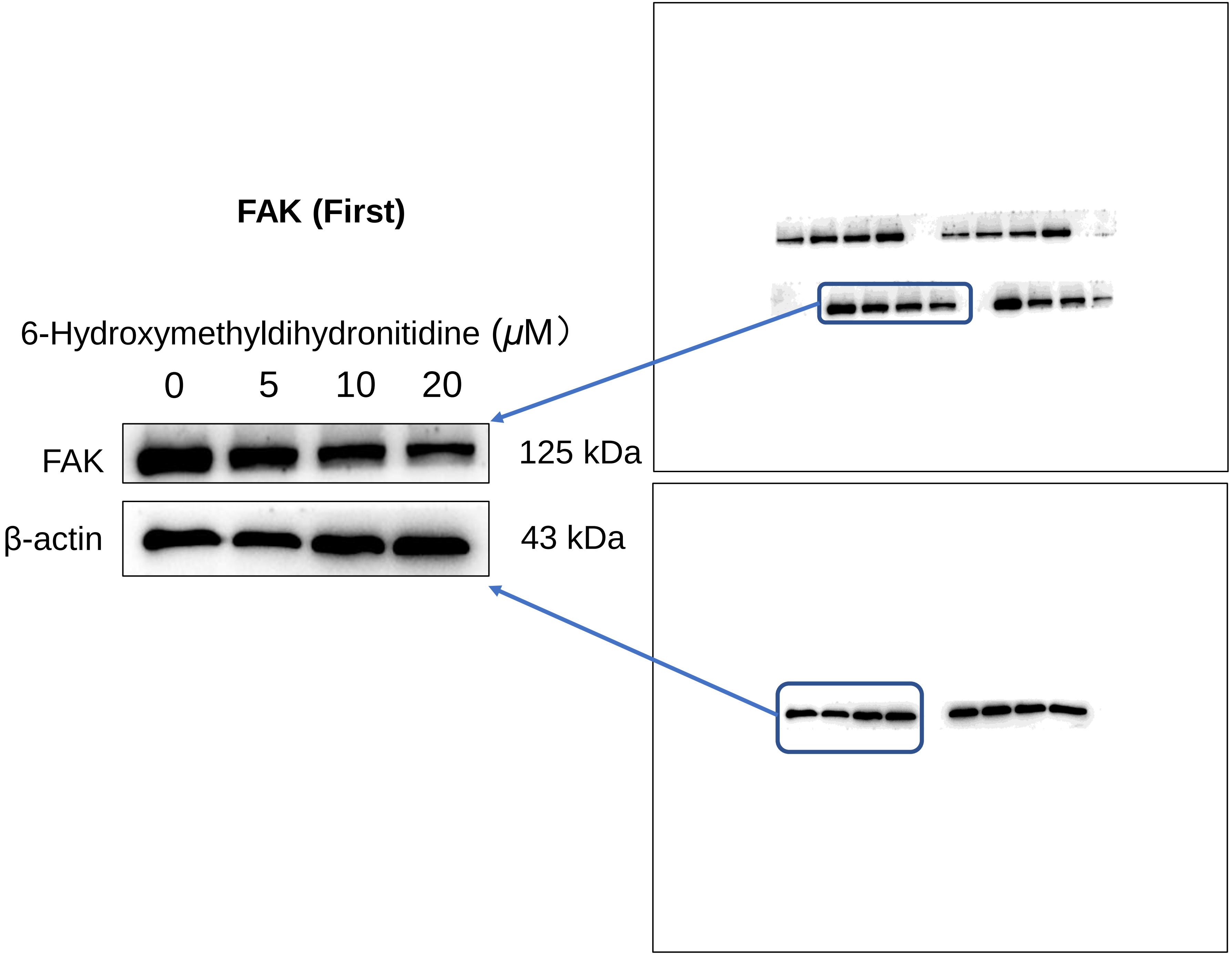

FAK (First)
6-Hydroxymethyldihydronitidine (μM)
0
5
10
20
FAK
125 kDa
β-actin
43 kDa

FAK (Second)

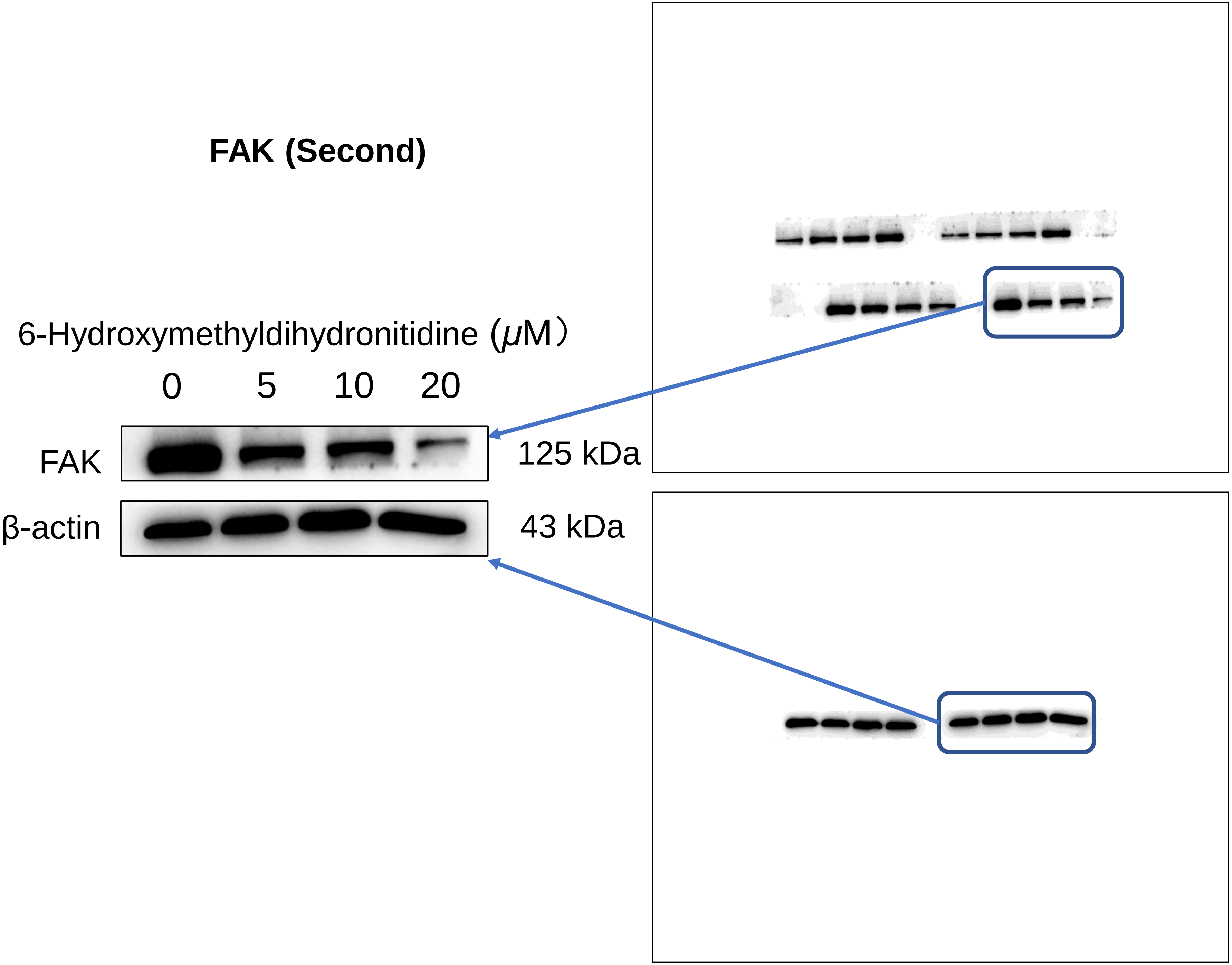
6-Hydroxymethyldihydronitidine (μM）
0
5
10
20
FAK
β-actin
125 kDa
43 kDa

FAK (Third)

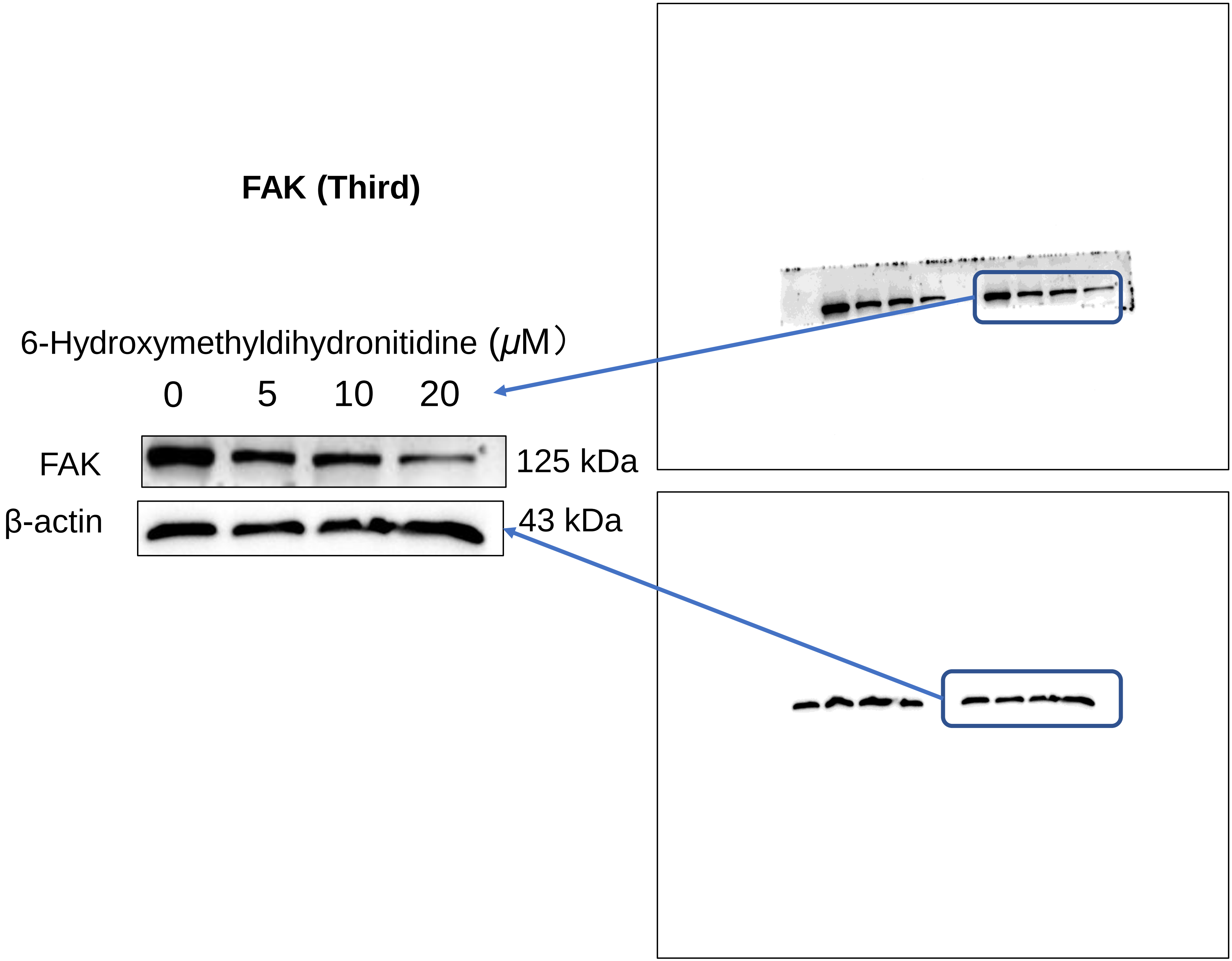
6-Hydroxymethyldihydronitidine (μM)
0
5
10
20
FAK
125 kDa
β-actin
43 kDa

p- FAK (First)

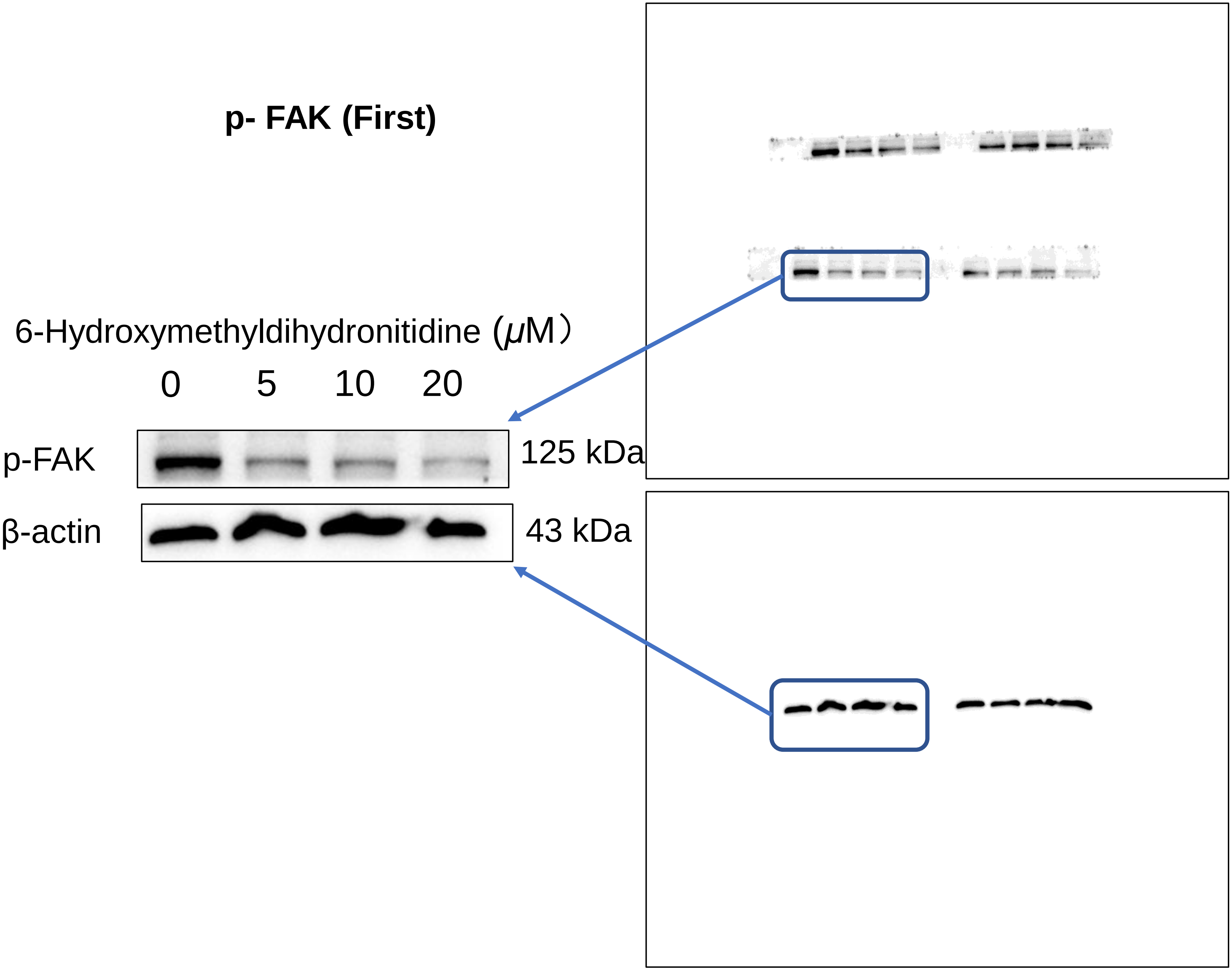
6-Hydroxymethyldihydronitidine (μM）
0
5
10
20
p-FAK
125 kDa
β-actin
43 kDa

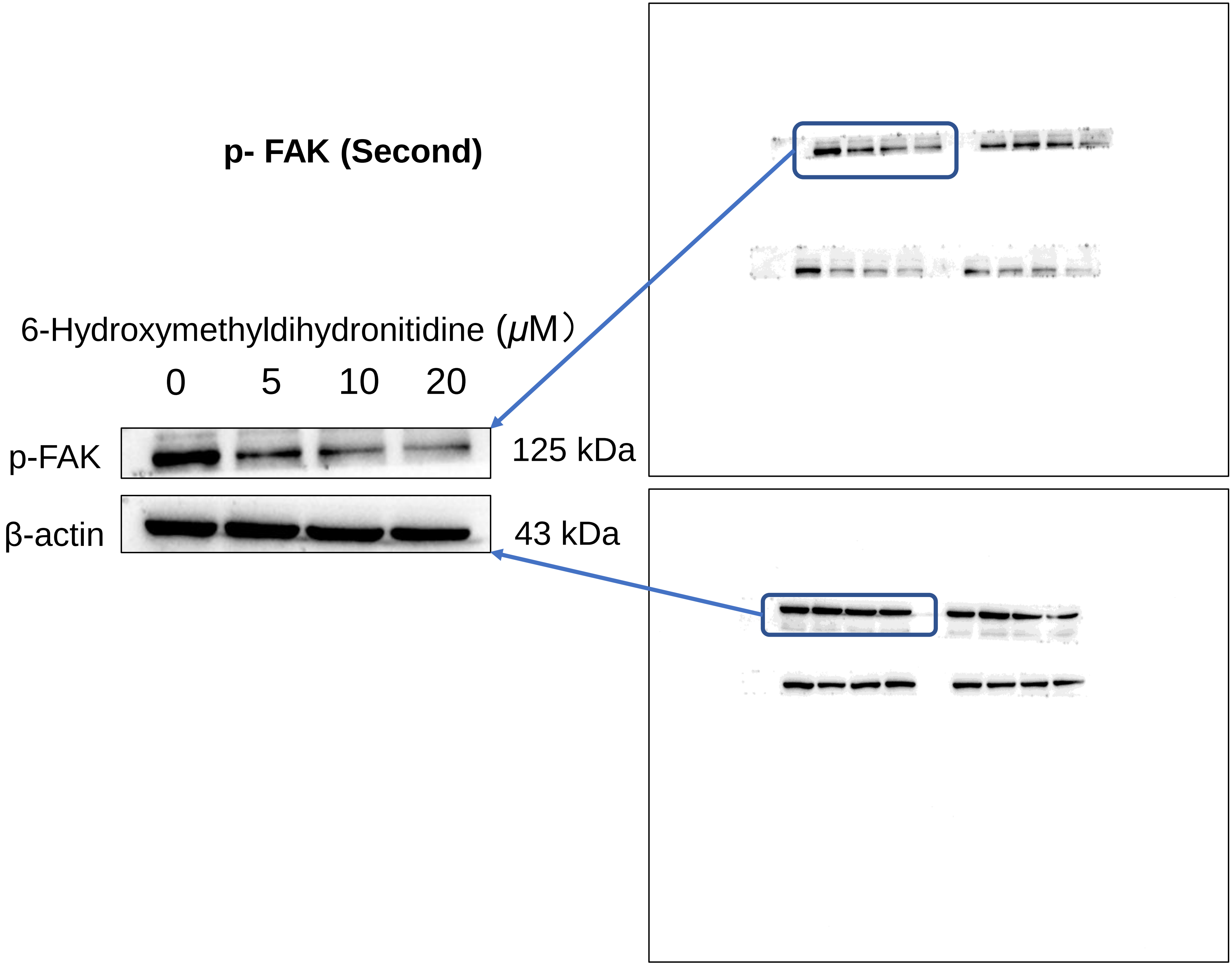
p- FAK (Second)
6-Hydroxymethyldihydronitidine (μM）
0
5
10
20
p-FAK
125 kDa
β-actin
43 kDa

p- FAK (Third)

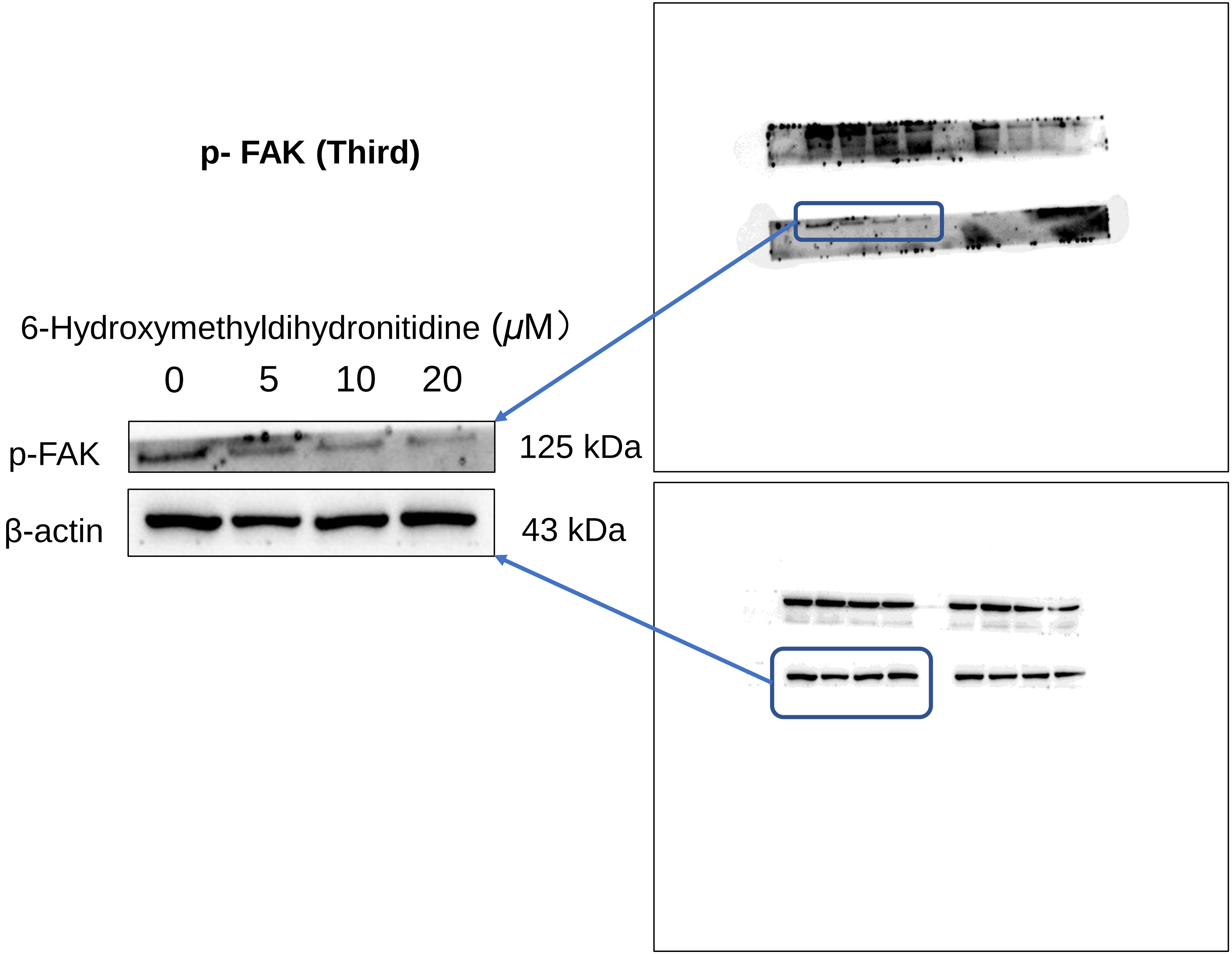

6-Hydroxymethyldihydronitidine (μM)
0
5
10
20
p-FAK
125 kDa
β-actin
43 kDa

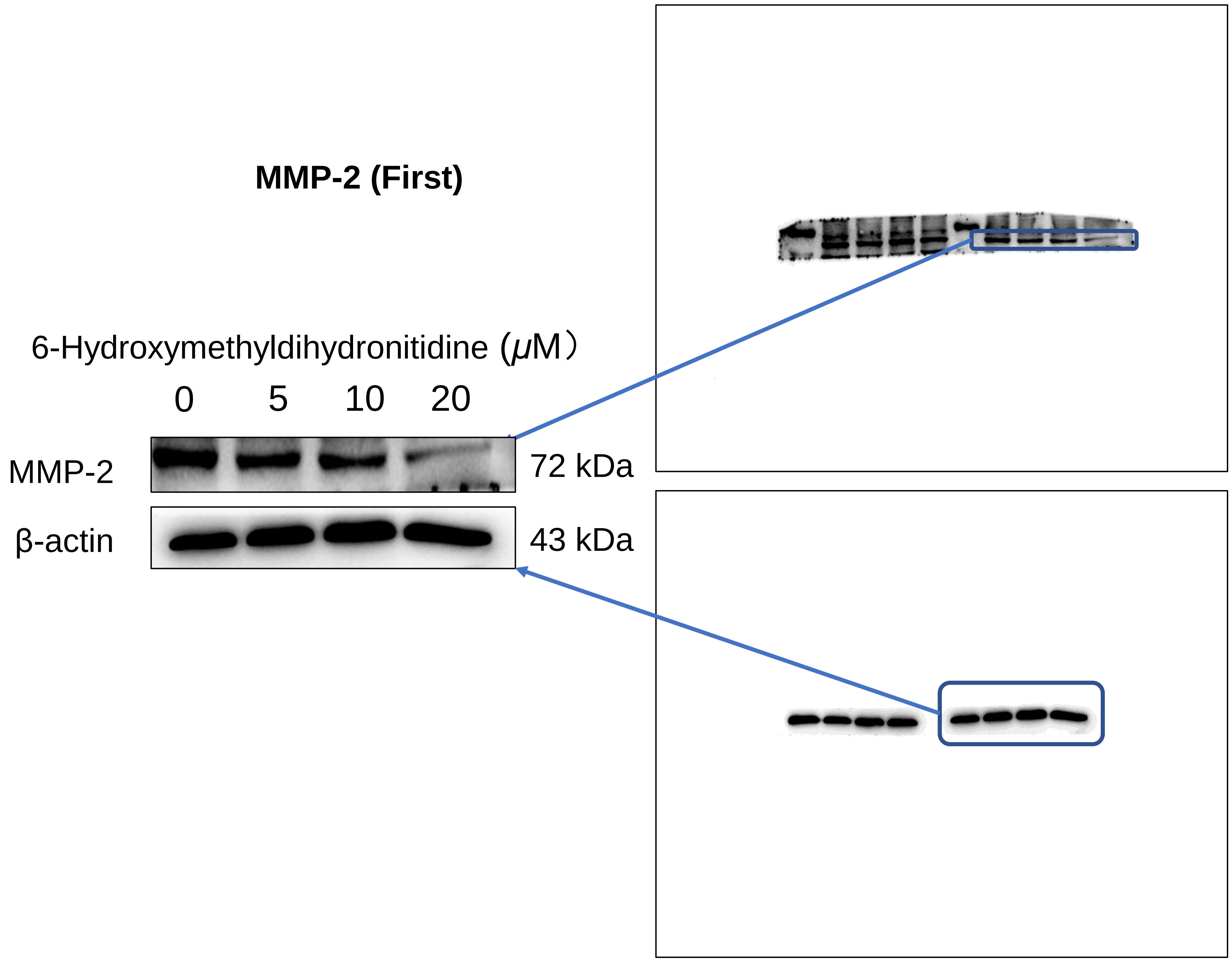
MMP-2 (First)
6-Hydroxymethyldihydronitidine (μM）
0
5
10
20
MMP-2
β-actin
72 kDa
43 kDa

MMP-2 (Second)

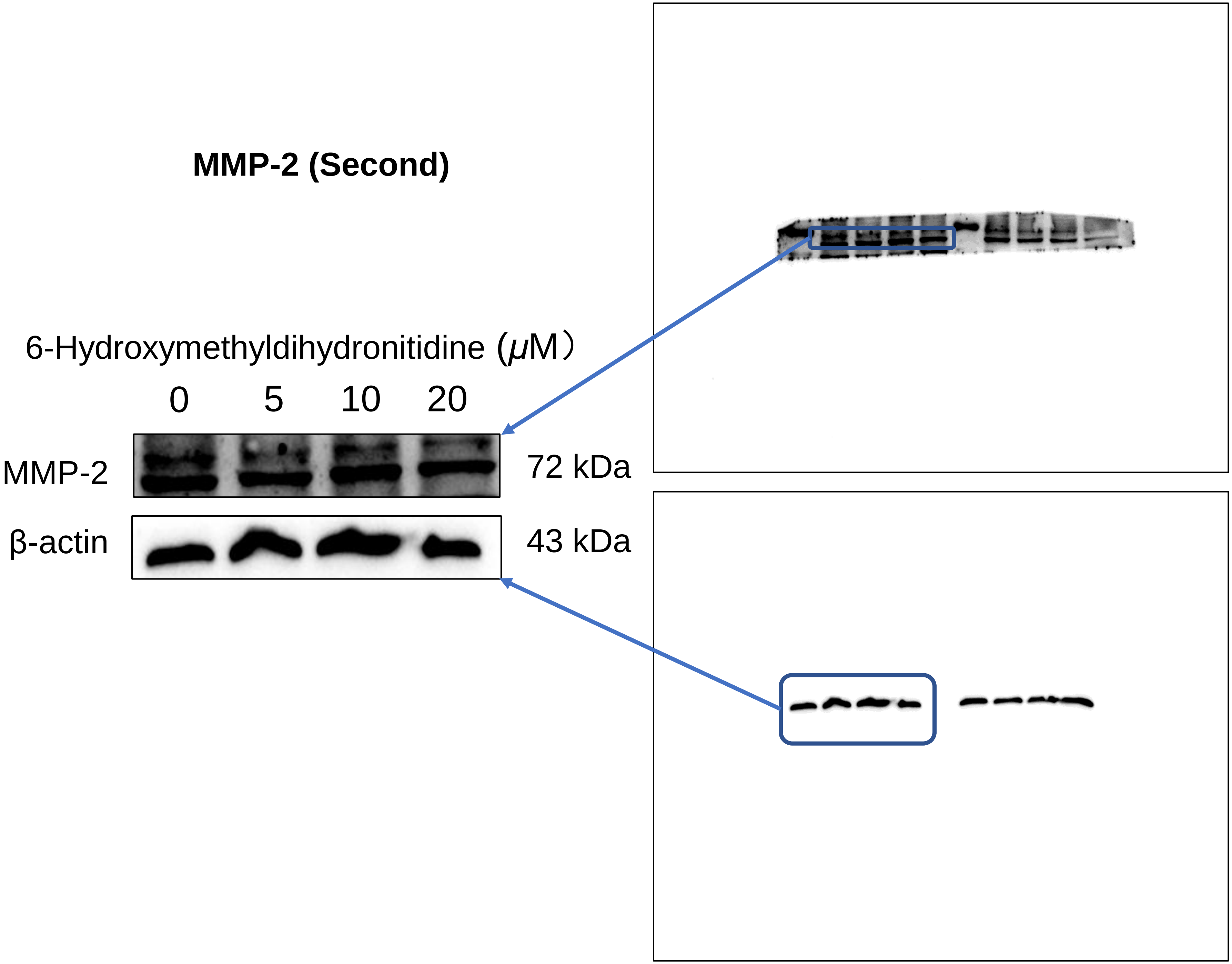
6-Hydroxymethyldihydronitidine (μM)
0
5
10
20
MMP-2
72 kDa
β-actin
43 kDa

MMP-2 (Third)

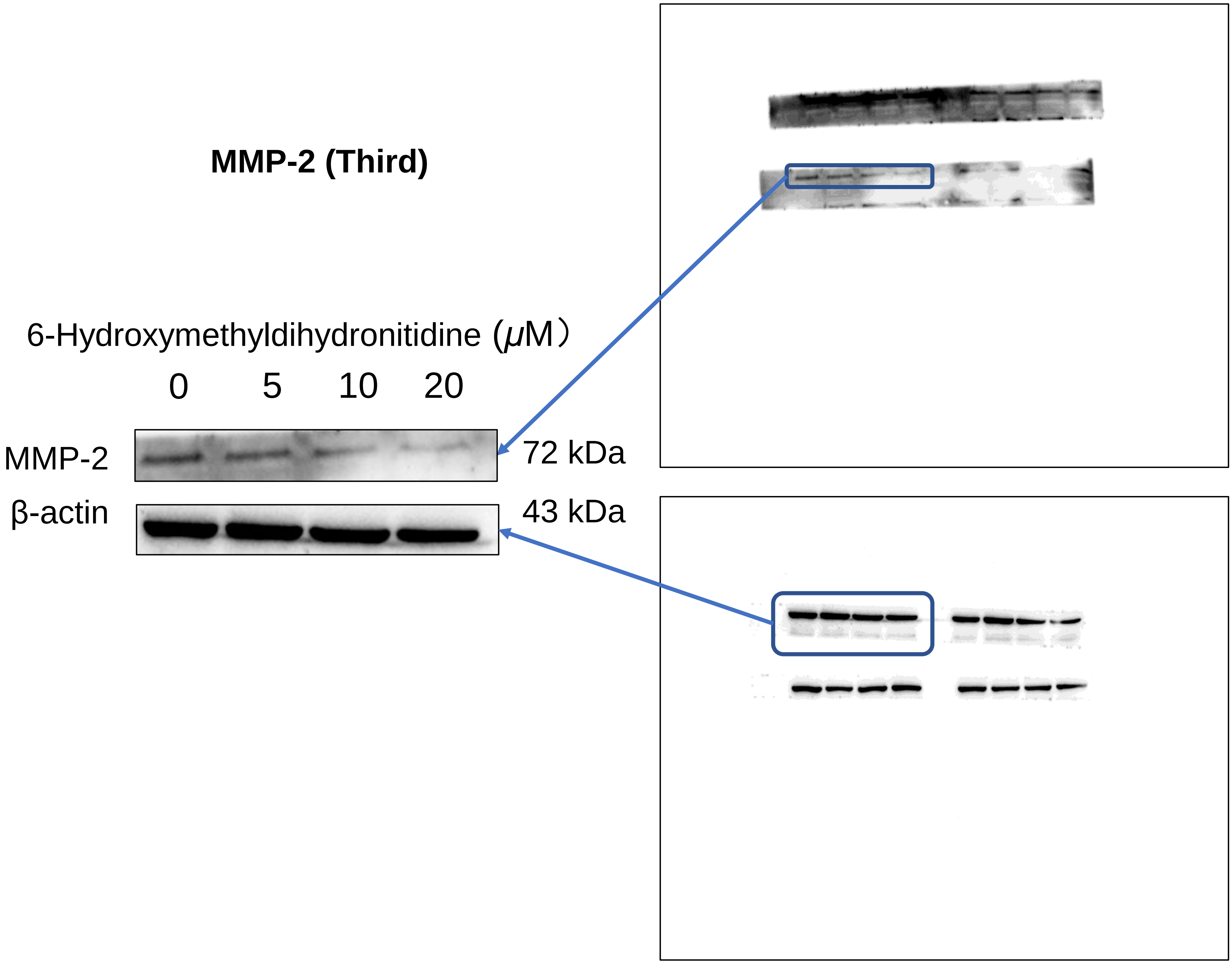

6-Hydroxymethyldihydronitidine (μM)
0
5
10
20
MMP-2
72 kDa
β-actin
43 kDa
