# Supplementary material for: A Natural Alkaloid, 6-Hydroxymethyldihydronitidine, Suppresses Tumor Progression by Co-Regulating Apoptosis, Ferroptosis, and FAK Pathways
Source: Biomolecules. 2025 Jun 4;15(6):814. doi: 10.3390/biom15060814 (PMC12190358; doi:10.3390/biom15060814)
Supplement: Supplementary file 1 [file biomolecules-15-00814-s001.zip › Supplementary Materials.pdf]

Supplementary Materials: A Natural Alkaloid, 6-Hydroxymethyldihydronitidine, Suppresses Tumor Progression by Co-regulating Apoptosis, Ferroptosis, and FAK Pathways

1. Detailed information on materials and reagents

Dulbecco's Modified Eagle's Medium (DMEM, VivaCell, Catalog No.: C3113-0500) and Fetal Bovine Serum (FBS, BI, Israel, Catalog No.: ST30-3302) were supplied by Lab Biotech Co., Ltd. (Jinan, China). The 3-(4,5-dimethylthiazol-2-y1)-2,5-diphenyl tetrazolium bromide (MTT) was purchased from BioFroxx (Guangzhou, China, Catalog No. 1334GR005). C11 BODIPY $^{581/591}$ was acquired from GlpBio (Shanghai, China, Catalog No. GC40165), and the CheKine™ Micro Reduced Glutathione (GSH) Assay Kit was purchased from Akkbine Biotechnology Co., Ltd. (Wuhan, China, Catalog No. KTB1600). Additionally, various assay kits, including the Annexin V-FITC Apoptosis Assay Kit (Catalog No. MA0220-2), Reactive Oxygen Species Assay Kit (Catalog No. S0033S), and BCA Protein Concentration Assay Kit (Catalog No. P0012), were obtained from Beyotime Biotechnology Co., Ltd. (Shanghai, China). The cell tracer CM-DiI was provided by Yeasen Biotechnology Co., Ltd. (Shanghai, China, Catalog No. 40792ES50). For immunoblotting, mouse monoclonal antibodies against STAT3 (Catalog No. 9139S), caspase-9 (Catalog No. 9508S), and rabbit monoclonal antibodies against cleaved caspase-3 (Catalog No. 9664S), caspase-3 (Catalog No. 9662S), Bcl-2 (Catalog No. 4223S), phospho-STAT3 (Tyr 705, Catalog No. 9145S), FAK (Catalog No. 3285S), phospho-FAK (Tyr 397, Catalog No. 8556S), and β-actin (Catalog No. 4970S) were purchased from Cell Signaling Technology (Danvers, MA, USA). Finally, goat anti-rabbit IgG-HRP (Catalog No. abs20040) and goat anti-mouse IgG-HRP (Catalog No. abs20001) were supplied by Univ Biotechnology Co., Ltd. (Shanghai, China).

2. Toxicity of 6-hydroxymethyldihydronitidine (6-HMDN) to zebrafish

2.1. Methods for toxicity assay of 6-HMDN

The toxicity assay of 6-HMDN was performed using normal zebrafish embryos. At 6 h post-fertilization, the embryos were collected and randomly divided into four groups (20/group). The experimental groups were treated with 0.05, 0.1, and 0.2 μM 6-HMDN for 72 h, while the control group received no treatment. The morphology of zebrafish embryos was observed under the microscope, and the survival rate was counted to evaluate the toxicity of 6-HMDN.

2.2. Non-toxic effects of 6-HMDN on normal zebrafish embryos

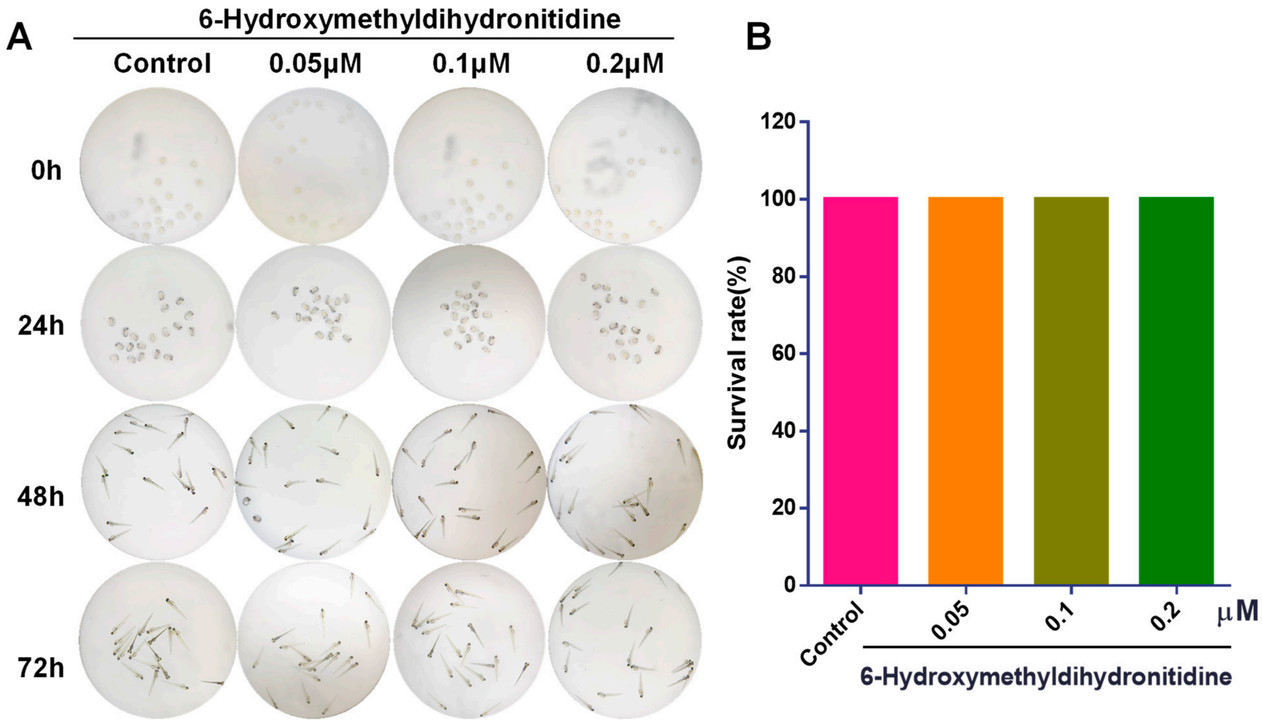

Figure S1. Toxic effects of 6-HMDN on zebrafish embryos.

3. NMR spectra of 6-HMDN

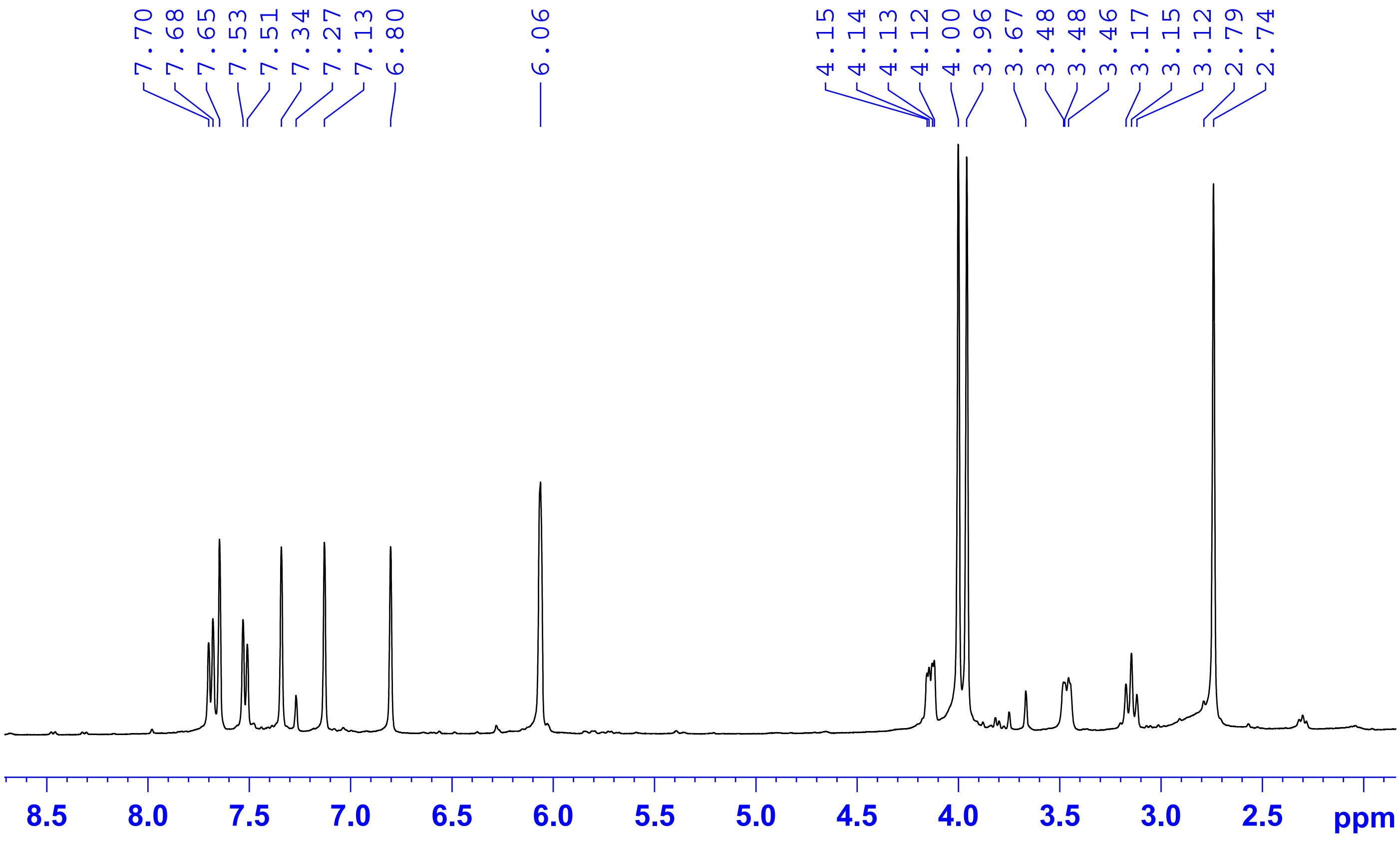

Figure S2. ^{1}H NMR spectrum of 6-HMDN.

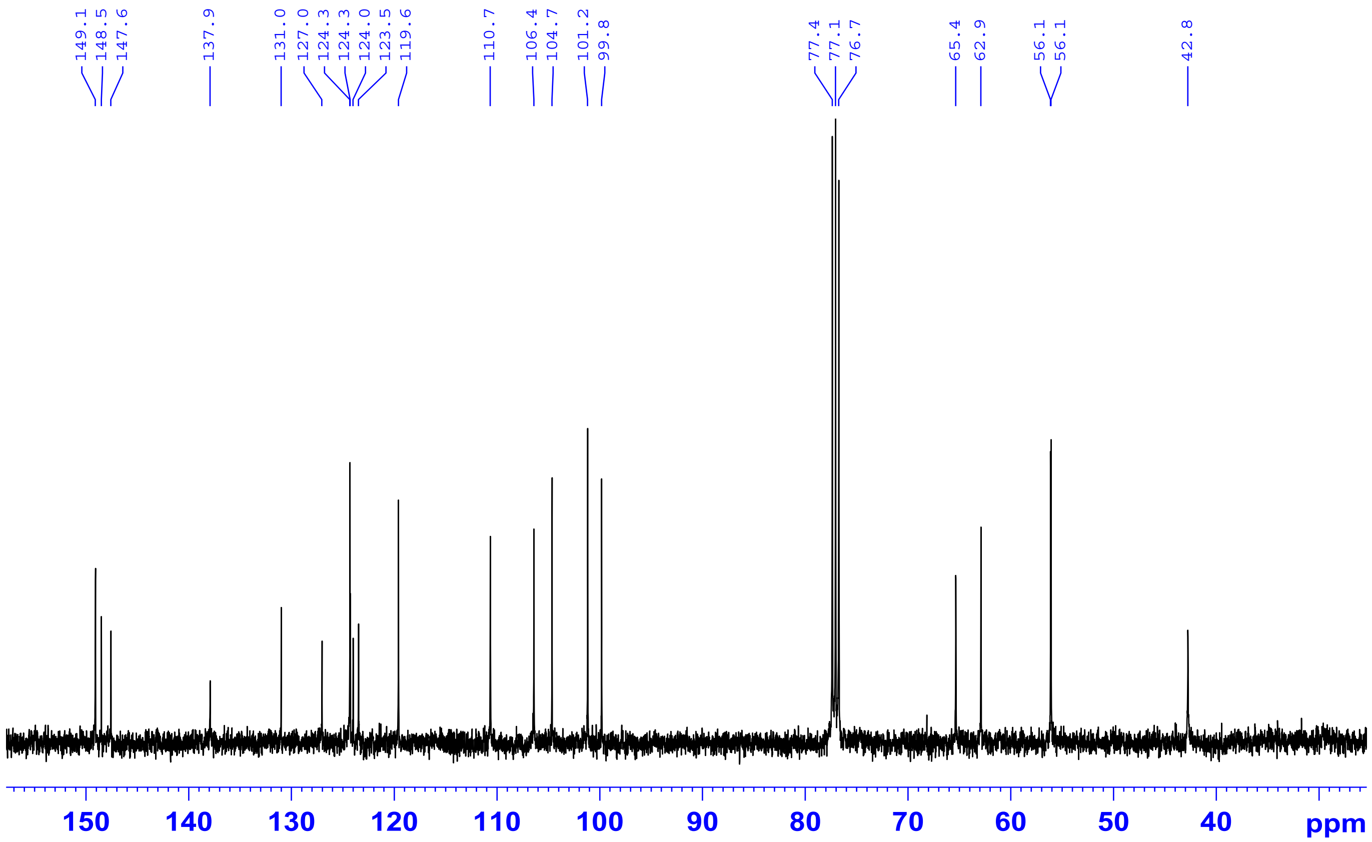

Figure S3. ^{13}C NMR spectrum of 6-HMDN.

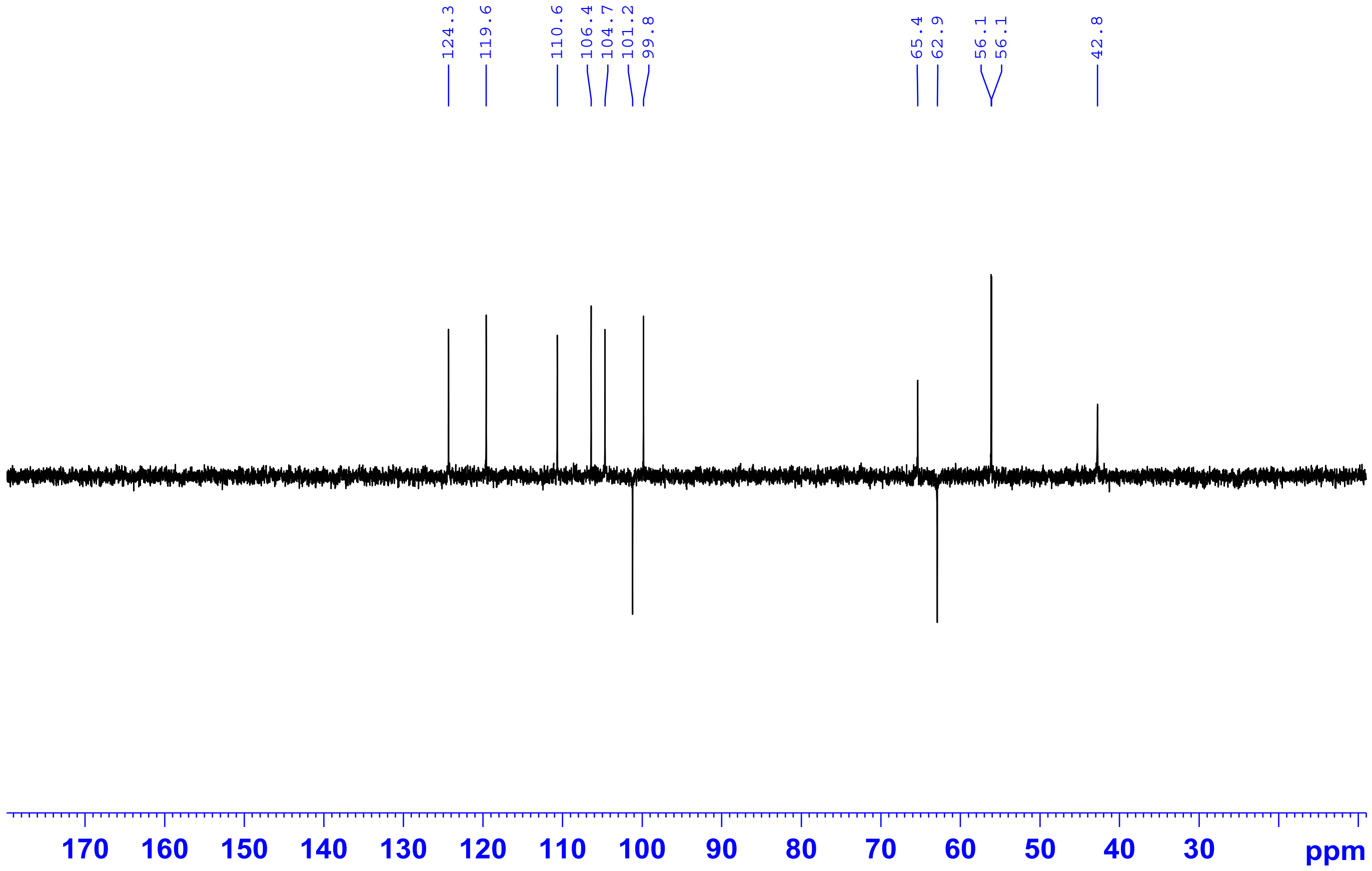

Figure S4. DEPT spectrum of 6-HMDN.
